# Pharmacoinformatic study of inhibitory potentials of selected flavonoids against papain-like protease and 3-chymotrypsin-like protease of SARS-CoV-2

**DOI:** 10.1186/s40816-022-00347-y

**Published:** 2022-09-08

**Authors:** Habibu Tijjani, Adegbenro P. Adegunloye, Auwalu Uba, Joseph O. Adebayo, Gideon A. Gyebi, Ibrahim M. Ibrahim

**Affiliations:** 1 Department of Biochemistry, Bauchi State University, Gadau, Nigeria; 2 Department of Biochemistry, University of Ilorin, Ilorin, Nigeria; 3 Present Address: Department of Microbiology, Bauchi State University, Gadau, Nigeria; 4 Department of Microbiology, Abubakar Tafawa Balewa University, Bauchi, Nigeria; 5 Department of Biochemistry, Bingham University, Karu, Nasarawa, Nigeria; 6 Department of Biophysics, Faculty of Sciences, Cairo University, Giza, Egypt

**Keywords:** Flavonoids, SARS-CoV-2, Papain-like protease, 3-Chymotrypsin-like protease, In silico

## Abstract

**Background:**

Inhibition of papain-like protease (PLpro) and 3-chymotrypsin-like protease (3CLpro) of severe acute respiratory syndrome coronavirus 2 (SARS-CoV-2) is projected to terminate its replication. Hence, these proteases represent viable therapeutic targets.

**Methods:**

Sixty-one flavonoids with reported activities against other RNA viruses were selected and docked in PLpro and 3CLpro. Flavonoids with better binding energies compared to reference inhibitors (lopinavir and ritonavir) in their interaction with PLpro and 3CLpro were selected for drug-likeness and ADMET analysis. The best representative flavonoid for each protease from the ADMET filtering analysis was subjected to molecular dynamics simulations (MDS) and clustering analysis of the trajectory files.

**Results:**

Licorice, ugonin M, procyanidin, silymarin, and gallocatechin gallate had better binding energies (-11.8, -10.1, -9.8, -9.7 and -9.6 kcal/mol respectively) with PLpro compared to lopinavir and ritonavir (-9.1 and -8.5 kcal/mol respectively). Also, isonymphaeol B, baicalin, abyssinone II, tomentin A, and apigetrin had better binding energies (-8.7, -8.3, -8.2, -8.1, and -8.1 kcal/mol respectively) with 3CLpro compared to lopinavir and ritonavir (-7.3 and -7.1 kcal/mol respectively). These flavonoids interacted with the proteases via hydrogen and non-hydrogen bonding. Of these flavonoids, silymarin and isonymphaeol B demonstrated most favourable combination of attributes in terms of binding energies, compliance with Lipinski rule for drug-likeness and favourable pharmacokinetics in silico. These two flavonoids exhibited appreciable degree of structural stability, maintaining strong interaction with residues in the different representative clusters selected during the MDS run.

**Conclusion:**

Silymarin and isonymphaeol B are proposed for further studies as compounds with potential activities against SARS-CoV-2.

**Supplementary Information:**

The online version contains supplementary material available at 10.1186/s40816-022-00347-y.

## Highlights

• Flavonoids displayed varying affinities for PLpro and 3CLpro of SARS-CoV-2

• They interacted via hydrogen and non-hydrogen bonds; nine and twenty-seven flavonoids had better binding affinities for PLpro and 3CLpro respectively than lopinavir and ritonavir

• Silymarin and isonymphaeol B demonstrated most favourable combination of attributes in terms of binding energies, compliance with Lipinski rule for drug-likeness and favourable pharmacokinetics.

• Silymarin and isonymphaeol B exhibited appreciable degree of structural stability, maintaining strong interaction with residues in the different representative clusters selected during the MDS run.

**Supplementary Information:**

The online version contains supplementary material available at 10.1186/s40816-022-00347-y.

## Introduction

Coronaviruses are a family of viruses that cause a wide range of diseases including common cold, severe acute respiratory syndrome (SARS), etc. The novel coronavirus, severe acute respiratory syndrome coronavirus-2 (SARS-CoV-2), shares about 80% and 50% homology with severe acute respiratory syndrome coronavirus (SARS-CoV) and Middle East respiratory syndrome coronavirus (MERS-CoV) respectively [1, 2]. The virus, SARS-CoV-2, is an enveloped positive-sense, single-stranded RNA virus, with genome size of about 30 kb [1]. It has crown-like spike protein on its membrane, and uses this to bind cell receptor, thereby facilitating virus–host cell membrane fusion and infection of target human cells [3]. The genetic content of the virus is then emptied into the host cell where the translation machinery of the host cell is used to make viral polyproteins. The proteolytic processing of this polyproteins yields structural and non-structural proteins. The non-structural proteins include papain-like protease (PLpro), 3-chymotrypsin-like protease (3CLpro), helicase and RNA-dependent RNA polymerase, among others [4]. The proteolytic processing of the polyproteins is performed by the viral cysteine proteases to yield 16 non-structural proteins; the PLpro cleaves first three sites at the N-terminus while the other protease, 3CLpro, cleaves and modify the viral polyproteins at 11 other sites [5, 6]. In addition to this proteolytic activity, PLpro reverses cellular ISGylation and ubiquitination processes, and may deubiquinate some host cell proteins, like interferon factor 3 and NF-κB, to suppress host innate immune system [7] and aid survival of the virus. Hence, both 3CLpro and PLpro are viable therapeutic targets.

Drugs such as remdesivir and chloroquine have been reported to be potent inhibitors of SARS-CoV-2 in vitro, and have been proposed for the treatment of the virus [8]. Furthermore, remdesivir has been reported previously to inhibit zoonotic CoV prior to emergency of SARS-CoV-2 [9]. Following remdesivir and chloroquine, several drugs and natural compounds have been repurposed for treatment of SARS-CoV-2, to facilitate accelerated drug discovery for the pandemic [10–14].

Number of cases and death associated with coronavirus disease 2019 (COVID-19) remains a concern. Hence, the need for alternative therapy against the causative agent, SARS-CoV-2. The use of natural products for the treatment of diseases is a practice that has been in existence since ancient times and their activities are attributed to the secondary metabolites present in them [15]. Flavonoids are secondary metabolites present in diverse plant species. They play a wide range of physiological roles in plants and many reports have indicated their pharmacological activities against infectious diseases, metabolic disorders and degenerative diseases. Hence, they are used as antibacterial, antifungal, anti-inflammatory and antiviral agents [16–19]. Flavonoids have been reported to be active against bacteria, such as Mycobacterium tuberculosis, and viruses, such as Streptococcus pneumonia, influenza virus and zika virus [19–22]. Among the benefits of antiviral activities of flavonoids is their several mechanisms by which they inhibit and act on the viruses. Flavonoids acts at multiple stages of viral infection, targeting their attachment, entrance, obstruct phases of viral DNA replication, translation of proteins, poly-protein processing and could inhibit the release of viruses from invasion other healthy host cells [23, 24]. Flavonoids are naturally occurring, ubiquitously in plants and major secondary metabolites [25]. Thus, the use of flavonoids against COVID-19 could prove to be a treatment option that is accessible, of low cost, and with little or no adverse effect on infected individuals. 5,7-dimethoxyflavanone-40-O-b-d-glucopyranoside, baicalin, Euchresta flavanone A, flemiflavanone D, hesperidin, kaempferol, luteolin, myricetin 3-rutinoside, naringen, quercetin-3-O-rhamnoside, rhoifolin and rutin are among several flavonoids identified through computational studies as potent inhibitors of SARS-CoV-2 [26–30]. Further studies, through combinatorial molecular simulations, ADMET analysis, and hybrid QM/MM approaches has identified some mechanism of action of the flavonoids against SARS-CoV-2, and their amino acid interactions in the binding pockets of SARS-CoV-2 structural and none structural proteins. Compounds which are active against SARS-CoV-2 proteases are expected to cause inhibition of the enzymes. Compounds that inhibit the viral proteases PLpro and 3CLpro could terminate the replication process of SARS-CoV-2. This study was, therefore, embarked on to evaluate the interaction of selected flavonoids with SARS-CoV-2 PLpro and 3CLpro and their pharmacokinetic parameters in silico.

## Methods

### Ligand preparation

Flavonoids with reported in vitro or in vivo activities against Mycobacterium tuberculosis, influenza virus, zika virus, Streptococcus pneumonia were searched for [19–22, 31–36 and others]. Careful selection of these compounds yielded 61 flavonoids (Supplementary material, Tables S1 and S2), which were used for this study. The flavonoid structures meeting the inclusion criteria were downloaded in SDF format from PubChem database (www.pubchem.ncbi.nlm.nih.gov). The compounds were converted to mol2 chemical format using Open babel [37] and to pdbqt format, after setting appropriate torsion centre for each ligand.

### Protein preparation

The crystal structures for SARS-CoV-2 papain-like protease (PLpro) and 3-chymotrypsin-like protease (3CLpro) with PDB code 6W9C and 6Y2E respectively were retrieved from the protein databank (www.rcsb.org). They were prepared by removing existing ligands and water molecules (if any), while missing polar hydrogen atoms were added using Autodock v4.2 program (Scripps Research Institute). Thereafter, non-polar hydrogen atoms were merged while polar hydrogen atoms were added to each enzyme. They were saved in dockable pdbqt format for molecular docking studies.

### Molecular docking

The molecular docking studies of the ligands (61 flavonoids and 2 reference compounds) and protein targets were carried out using Autodock Vina [38]. The binding energies of each compound were recorded and used in rating the compounds for which PLpro and 3CLpro of SARS-CoV-2 had highest affinities. Flavonoids that displayed lower binding energies (Kcal/mol) were considered to be those for which the enzymes had higher binding affinities. Thereafter, the molecular interactions of the ligands with each protein were viewed with Discovery Studio Visualizer, BIOVIA, 2016.

### Studies on physicochemical properties and ADMET

The flavonoids with best docking parameters against each protein were virtually screened for their physicochemical properties and absorption, distribution, metabolism, elimination, and toxicity (ADMET) properties. Webservers, SwissADME (http://www.swissadme.ch/index.php) [39] and ADMETlab (http://admet.scbdd.com/calcpre/index), were used for the predictive analysis. The Lipinski’s rule of five [40] was used to screen for ligands with drug-likeness.

### Molecular dynamic simulation (MDS) and clustering

The best representative flavonoids for each protease from the docking and ADMET filtering analysis were subjected to Molecular Dynamic simulation (MDS) analysis using NAMD version 2.13. From the trajectories files of the apo protease and best complexes (PLpro-Silymarin and 3CLpro -Isonymphaeol B), various structural parameters were calculated as a function of time to explore the structural behaviour of the proteins, and protein–ligand complexes [41]. Necessary files for MDS were generated using CHARMM-GUI webserver [42, 43]. For each complex or apo protein, the system was minimized, using steepest descent algorithm, in constant number of atoms, constant volume and constant temperature (NVT) ensemble then was used in a production run for 100 ns in NVT ensemble. The concentration of ions and temperature were set to be 0.154 M NaCl and 310 K, respectively. Backbone-Root Mean Square Deviation (RMSD), Per residue Root Mean Square Fluctuations (RMSF), Radius of Gyration (RoG), Surface Accessible Surface Area (SASA) were calculated using VMD TK console scripts to analyze the trajectories [44]. Complexes were clustered using TTClust version 4.7.2 which utilizes elbow method to determine the optimal number of clusters [45]. For each cluster, a representative frame was produced and was further used in the analysis of interaction between the compounds and protein using Protein Ligand Interaction Profiler (PLIP) webserver [46]. The interactions between the compounds and protein images were generated using PyMol V 2.2.2 [47].

## Results

### Molecular docking and ligand interactions of Flavonoids against PLpro and 3CLpro

**Fig. 1:**
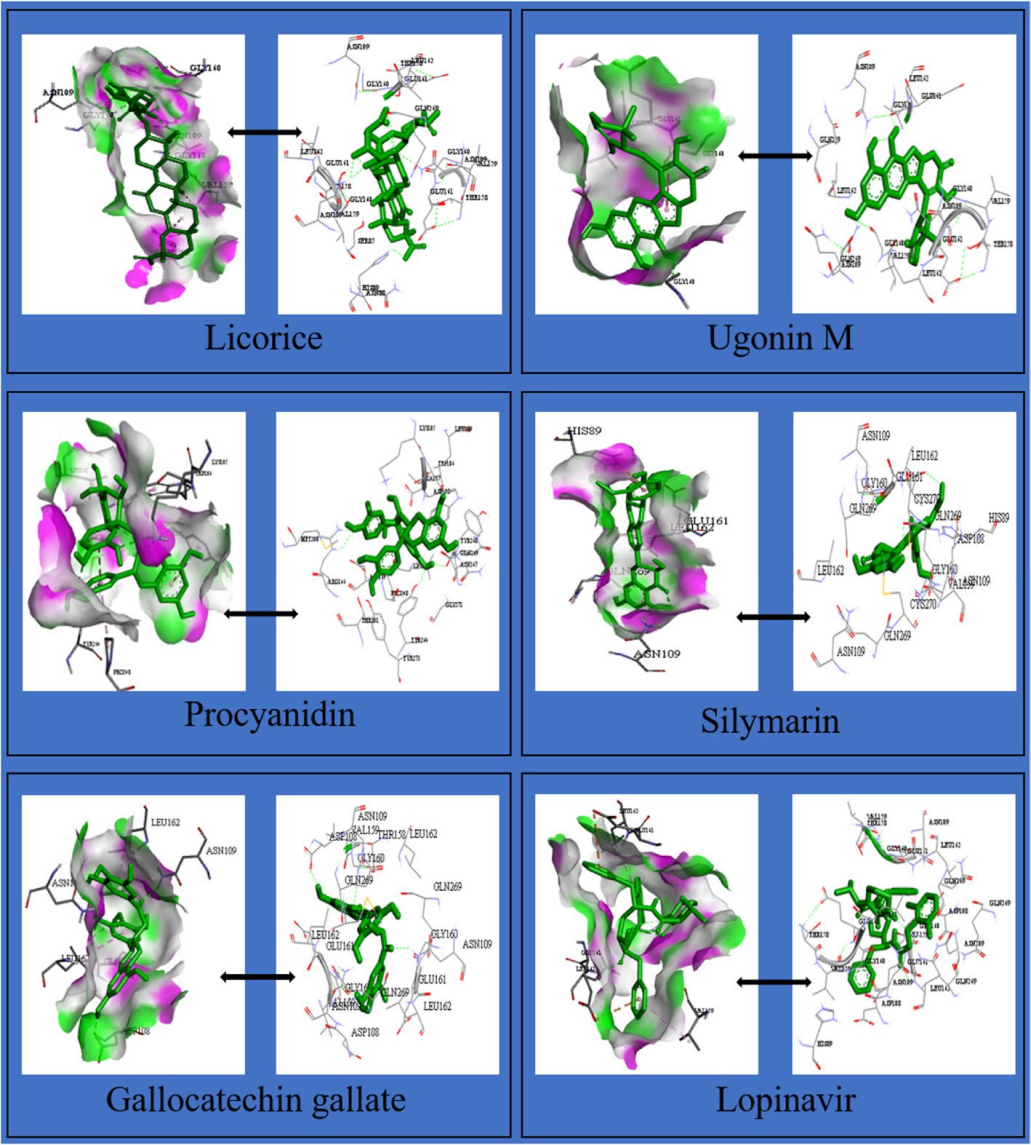
Interaction of selected flavonoids with amino acids of the binding pocket of PLpro of SARS-CoV-2

**Fig. 2:**
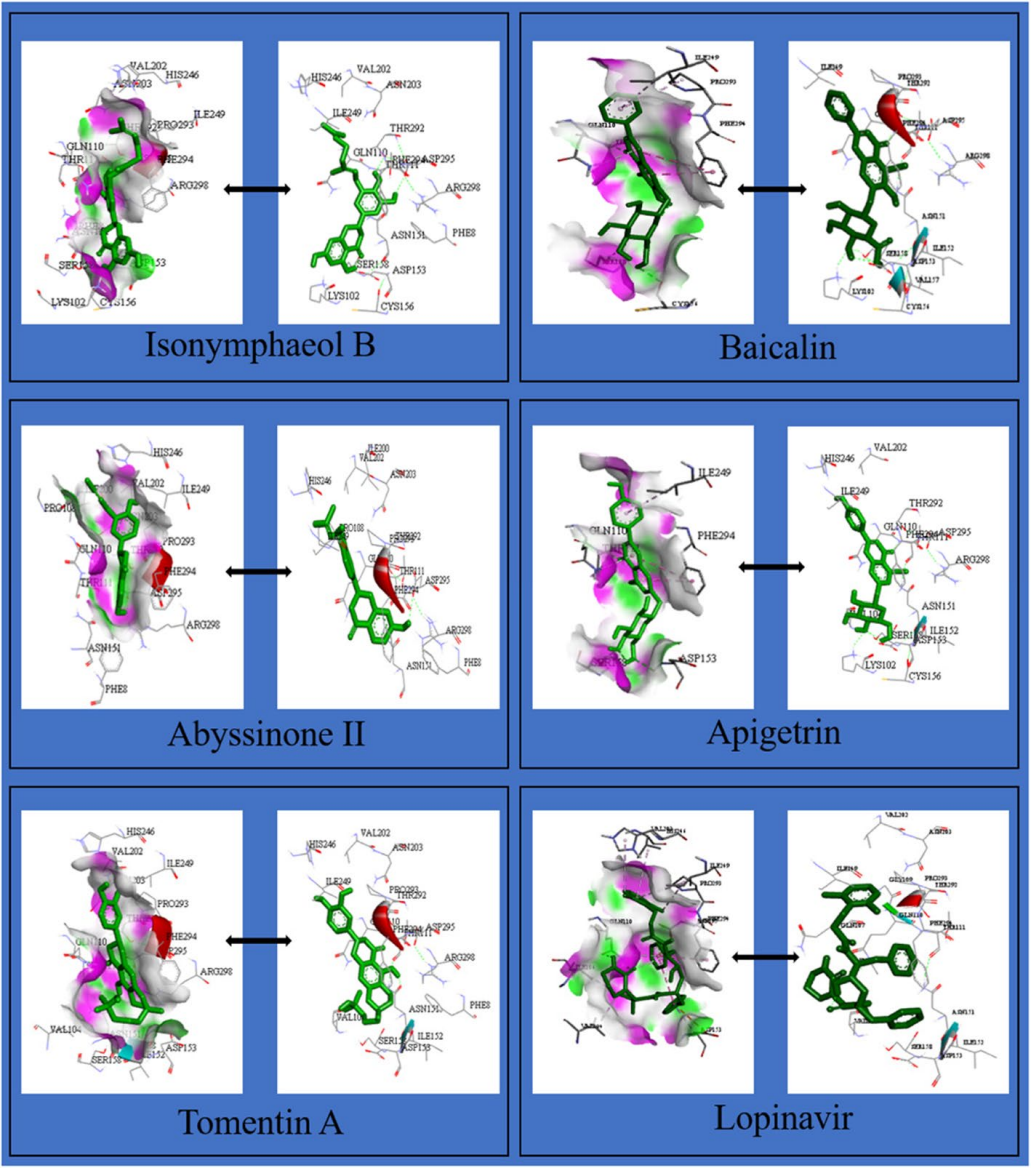
Interaction of selected flavonoids with amino acids of the binding pocket of 3CLpro of SARS-CoV-2

The binding energies of these flavonoids ranged from -11.8 to -7.0 kcal/mol for PLpro, and -8.7 to -6.1 kcal/mol for 3CLpro (Supplementary material, Tables S1 and S2). The binding affinities of PLpro for nine flavonoids were higher compared to lopinavir (-9.1 kcal/mol) while those for twenty-two flavonoids were higher compared to ritonavir (-8.5 kcal/mol; Supplementary material, Table S1). The binding affinities of 3CLpro for twenty-seven flavonoids were higher compared to lopinavir (-7.3 kcal/mol) while those for thirty-eight flavonoids were higher compared to ritonavir (-7.1 kcal/mol; Supplementary material, Table S2). The twenty flavonoids with best docking ratings for PLpro and 3CLpro are presented in Table 1 and Table 2 respectively. The interactions of licorice, ugonin M, procyanidin, silymarin and gallocatechin gallate with PLpro had binding energies of -11.8, -10.1, -9.8, -9.7 and -9.6 kcal/mol respectively (Table 1) while isonymphaeol B, baicalin, abyssinone II, apigetrin, and tomentin A had binding energies of -8.7, -8.3, -8.2, -8.1, and -8.1 kcal/mol respectively for 3CLpro (Table 2). These ligands interacted with PLpro and 3CLpro via hydrogen and non-hydrogen bonding. From the best docked compounds, both PLpro and 3CLpro had higher binding affinities for seven flavonoids compared to the two reference compounds—lopinavir and ritonavir (Table 3). The Interaction of flavonoids with amino acids of the binding pocket of PLpro and 3CLpro for which the highest binding affinities were recorded are presented in Figs. 1 and 2.

**Table 1:** Binding Energies and Amino Acid Interactions of Flavonoids with Papain-like Protease (PLpro) of SARS-CoV-2^a^

**Compound(PubChem CID)**	**Hydrogen bonding–related residues**	**Non- hydrogen bonding–related residues**	**Binding Energy (Kcal/mol)**
Lopinavir*(92727)	LEU162	GLU161B, VAL159, GLU161A, LEU162	-9.1
Ritonavir*(392622)	-	GLY160	-8.5
Licorice(163463)	HIS89, ASN109B, GLY160A, ASN109C, GLY160C	HIS89, VAL159, GLY160	-11.8
Ugonin M(135891244)	GLY160A	LEU162B, GLY160B	-10.1
Procyanidin(107876)	TYR268, LYS105	TYR268, LEU162, TRP106, PRO248, TYR264	-9.8
Silymarin(5213)	ASN108C, GLN2691, GLU161A	HIS29B, LEU162A	-9.7
Gallocatechin gallate(5276890)	ASN109B, ASP108, GLN269, ASN109C	LEU162B, LEU162C	-9.6
Epigallocatechin gallate(65064)	ASN109C, GLN269, ASP108, ASN109B	LEU162C, LEU162B	-9.6
Isoquercetin(5280804)	VAL159, GLN269, GLN269	LEU162C, ASN109, LEU162A	-9.3
Isonymphaeol B(10070991)	--	GLU161B, LEU162B, LEU162A, VAL159B, HIS89B	-9.3
Baicalin(64982)	ASP108, LYS157, GLU161, LEU162, TYR264	TRP106, LYS105, ASP164, ASP108	-9.2
Hyperin(5281643)	GLU161, ASN109, GLN269	ASN109	-9.1
Flavopiridol(5287969)	THR158	GLY160, GLU161A, VAL159, LEU162, GLN269, GLU161C	-9.0
Apigetrin(5280704)	ASN109, GLN269, GLN269, LEU162	ASP108, LEU162	-8.9
Houttuynoid B(57412150)	LEU162	GLU161, GLY160	-8.9
Methoxymatteucin(158031)	VAL159, LEU162, GLY160	LEU162, VAL159, GLU161, HIS89	-8.8
Rutin(5280805)	VAL159A, ASP108A, ASN109C, LEU162A, GLY160B, VAL159B, LEU162B, GLY160A	GLU161A, GLY160C, GLU161C	-8.8
Astragalin(5282102)	ASP108, LEU162	LEU162C, LEU162A, ASN109	-8.7
Kaempferol-3-O-glucorhamnoside(5318761)	LEU162A, LEU162B, ASN109	ASN109, VAL159, GLY160, GLU161	-8.7
Tomentin A(71659627)	ASN109C, GLY160C	HIS89A, GLU161C, LEU162C	-8.7
Tomentin B(71659628)	ASN109C	GLU161, LEU162	-8.7
Abyssinone II(10064832)	ASN109	LEU162	-8.6

^*^Reference compounds**;**.^a^Results presented here are for the twenty flavonoids for which the enzyme has the highest binding affinities; those for other flavonoids are presented in Supplementary material, Table S1

**Table 2:** Binding energies and amino acid interactions of flavonoids with 3-chymotrypsin-like protease (3CLpro) of SARS-CoV-2^a^

**Compound(PubChem CID)**	**Hydrogen bonding–related residues**	**Non- hydrogen bonding–related residues**	**Binding Energy (Kcal/mol)**
Lopinavir*(92727)	GLN110	ILE106, VAL104, PHE294, ASP295, ILE249, PRO293, HIS246, VAL202, ASP153	-7.3
Ritonavir*(392622)	GLN110	GLN110, ARG105, PHE103, VAL104, VAL202, PRO132, ILE200, HIS246, PHE294	-7.1
Isonymphaeol B(10070991)	THR292, ASP295, ARG298, SER158	THR111, PHE294, ASP153, PRO293, ILE249, VAL202, HIS246	-8.7
Baicalin(64982)	CYS156, THR111, SER158	PHE294, PRO293, ILE249, GLN110	-8.3
Abyssinone II(10064832)	ASP295	ILE249, HIS246, VAL202, ILE200, PRO293, PHE294, ARG298	-8.2
Apigetrin(5280704)	THR111, ASP153, SER158	PHE294, PRO293, GLN110	-8.1
Tomentin A(71659627)	ASN151, THR111	ILE249, PHE294	-8.1
Tomentin B(71659628)	ASN151, THR111	PHE294, ILE249	-8.1
Isobavachalcone(5281255)	ASN151	PRO293, PRO132, ILE200, HIS246	-8.0
Sanggenol A(15233693)	SER158	THR111, ILE249, HIS246, VAL202, PRO293, ASP153, PHE294	-8.0
Silymarin(5213)	--	VAL202, PHE294, ASN151, ASP295, ILE249, PRO108	-8.0
Rutin(5280805)	ASP295, GLY109	PHE294, PRO293, THR292, ILE249	-7.9
Methoxymatteucin(158031)	THR111	PRO108, ILE249, PRO293, PHE294	-7.8
3,4'-dihydroxyflavone(145726)	THR111	VAL202, ILE249, PRO293, THR292, PHE294	-7.7
Rhoifolin(5282150)	SER158, GLN110, THR111, ASP295, PHE294	SER158, PHE294, PRO108, PHE8, ASN151	-7.7
Licorice(163463)	GLN110	PHE294, HIS246, ILE249	-7.6
Gallocatechingallate(5276890)	ASN151, ASP153, LYS102	VAL104, PHE294, ASP153	-7.6
Procyanidin(107876)	ASP295, THR111, LYS102, ARG105	VAL104, ASP153	-7.6
Ugonin M(135891244)	GLY109	VAL202, ILE249, PRO293	-7.6
Baicalein(5281605)	ASN151, ASP295	ARG298, ILE249, GLN110, PRO293	-7.5
Diosmetin(5281612)	PRO108, THR111, ASN151	VAL202, PHE294, THR292, PRO293	-7.5
Flavopiridol(5287969)	THR111, ASP295	PHE294, ILE249, GLN110	-7.5

^*^Reference compounds**;**.^a^Results presented here are for the twenty flavonoids for which the enzyme has the highest binding affinities; those for other flavonoids are presented in Supplementary material, Table S2

**Table 3:** Flavonoids with highest binding affinities on interacting with both PLpro and 3CLpro of SARS-CoV-2

Compound	PubChem CID	Binding Energy (Kcal/mol) with PLpro	Binding Energy (Kcal/mol) with 3CL pro
Lopinavir^a^	92,727	-9.1	-7.3
Ritonavir^a^	392,622	-8.5	-7.1
Licorice	163,463	-11.8	-7.6
Ugonin M	135,891,244	-10.1	-7.6
Procyanidin	107,876	-9.8	-7.6
Silymarin	5213	-9.7	-8.0
Gallocatechingallate	5,276,890	-9.6	-7.6
Isonymphaeol B	10,070,991	-9.3	-8.7
Baicalin	64,982	-9.2	-8.3

^a^Reference compounds

### Physicochemical properties and ADMET of Flavonoids against PLpro and 3CLpro

The graphical summary of pharmacokinetic properties of top binding flavonoids with PLpro and 3CLpro are presented in Supplementary material (Fig. S1).

The physicochemical properties of the best binding flavonoids revealed that ugonin M, silymarin, isonymphaeol B, abyssinone II, apigetrin, and tomentin A had drug-like characteristics as predicted by Lipinski’s rule of five (Tables 4 and 5). The results revealed that gallocatechin gallate, baicalin and apigetrin were soluble in water, ugonin M, procyanidin, silymarin, abyssinone II and tomentin A were moderately soluble in water while licorice and isonymphaeol B were poorly soluble in water (Tables 4 and 5).

**Table 4:** In silico Physicochemical and ADMET^a^ Parameters of flavonoids with highest binding affinities on interacting with PLpro of SARS-CoV-2

Properties	Licorice	Ugonin M	Procyanidin	Silymarin	Gallocatechin gallate
Formula	C_42_H_62_O_16_	C_25_H_24_O_7_	C_30_H_26_O_13_	C_25_H_22_O_10_	C_22_H_18_O_11_
Molecular weight (g/mol)	822.93	436.45	594.52	482.44	458.37
Num. heavy atoms	58	32	43	35	33
Num. arom. heavy atoms	0	17	24	18	18
Fraction Csp3	0.86	0.32	0.20	0.24	0.14
Num. rotatable bonds	7	2	4	4	4
Num. H-bond acceptors	16	7	13	10	11
Num. H-bond donors	8	4	10	5	8
Molar Refractivity	202.84	123.23	147.52	120.55	112.06
XLOGP3	2.80	3.39	1.95	1.90	1.17
Lipinski violation*	3	0	3	0	2
**Water Solubility**					
Log *S* (ESOL)	-6.24	-4.94	-4.90	-4.14	-3.56
Solubility (mg/ml)	4.69 × 10^–04^	4.98 × 10^–03^	7.42 × 10^–03^	3.46 × 10^–02^	1.27 × 10^–01^
Class	Poorly soluble	Moderately soluble	Moderately soluble	Moderately soluble	Soluble
**ADMET**					
GI absorption	Low	Low	Low	Low	Low
BBB permeant	No	No	No	No	No
P-gp substrate	Yes	Yes	No	No	No
CYP1A2 inhibitor	No	No	No	No	No
CYP2C19 inhibitor	No	No	No	No	No
CYP2C9 inhibitor	No	Yes	No	No	No
CYP2D6 inhibitor	No	No	No	No	No
CYP3A4 inhibitor	No	No	No	Yes	No
Log *K*_p_ (cm/s) (skin permeation)	-9.33	-6.56	-8.54	-7.89	-8.27
hERG Channel Blockers	Yes	No	Yes	Yes	Yes
Ames Mutagenicity	Negative	Negative	Negative	Negative	Positive
H-HT	Negative	Positive	Negative	Negative	Negative

^a^ADMET: Absorption, distribution, metabolism, elimination, and toxicity; GI: Gastro-intestinal; BBB: Blood Brain Barrier; P-gp: permeability glycoprotein; CYP: cytochrome P450; hERG: human Ether-à-go-go-Related Gene; H-HT: Human Hepatotoxicity; *Number of rules violated out of the Lipinski’s rule of five

**Table 5:** In silico physicochemical and ADMET^a^ parameters of flavonoids with highest binding affinities on interacting with 3CLpro of SARS-CoV-2

Properties	Isonymphaeol B	Baicalin	Abyssinone II	Apigetrin	Tomentin A
Formula	C_25_H_28_O_6_	C_21_H_18_O_11_	C_20_H_20_O_4_	C_21_H_20_O_10_	C_25_H_30_O_7_
Molecular weight (g/mol)	424.49	446.36	324.37	432.38	442.50
Num. heavy atoms	31	32	24	31	32
Num. arom. heavy atoms	12	16	12	16	12
Fraction Csp3	0.32	0.24	0.25	0.29	0.48
Num. rotatable bonds	6	4	3	4	5
Num. H-bond acceptors	6	11	4	10	7
Num. H-bond donors	4	6	2	6	4
Molar Refractivity	120.87	106.72	93.27	106.11	120.61
XLOGP3	5.80	1.11	4.22	1.81	4.01
Lipinski violation^*^	0	2	0	0	0
**Water Solubility**					
Log *S* (ESOL)	-6.02	-3.41	-4.68	-3.78	-5.06
Solubility (mg/ml)	4.09 × 10^–04^	1.73 × 10^–01^	6.75 × 10^–03^	7.19 × 10^–02^	3.88 × 10^–03^
Class	Poorly soluble	Soluble	Moderately soluble	Soluble	Moderately soluble
**ADMET**					
GI absorption	High	Low	High	Low	High
BBB permeant	No	No	Yes	No	No
P-gp substrate	No	Yes	No	Yes	Yes
CYP1A2 inhibitor	Yes	No	Yes	No	No
CYP2C19 inhibitor	No	No	Yes	No	No
CYP2C9 inhibitor	Yes	No	Yes	No	No
CYP2D6 inhibitor	No	No	Yes	No	No
CYP3A4 inhibitor	Yes	No	Yes	No	No
Log *K*_p_ (cm/s) (skin permeation)	-4.77	-8.23	-5.28	-7.65	-6.15
hERG Channel Blockers	No	Yes	Yes	No	No
Ames Mutagenicity	Negative	Positive	Negative	Positive	Negative
H-HT	Negative	Negative	Positive	Positive	Negative

^a^ADMET: Absorption, distribution, metabolism, elimination, and toxicity; GI: Gastro-intestinal; BBB: Blood Brain Barrier; P-gp: permeability glycoprotein; CYP: cytochrome P450; hERG: human Ether-à-go-go-Related Gene; H-HT: Human Hepatotoxicity; *Number of rules violated out of the Lipinski’s rule of five

### Molecular dynamic simulation (MDS) and clustering of isonymphaeol B and silymarin on PLpro and 3CLpro

**Fig. 3:**
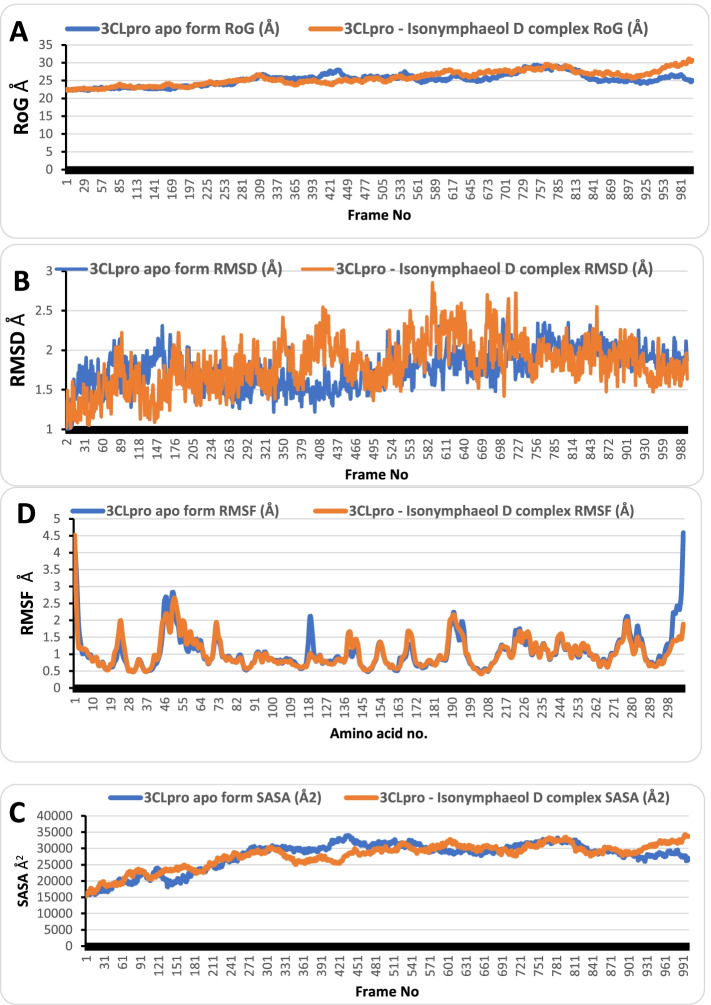
The SASA, RoG, RMSD, and RMSF of 3CLpro apo form (blue) and 3CLpro–Isonymphaeol B complex (orange)

**Fig. 4:**
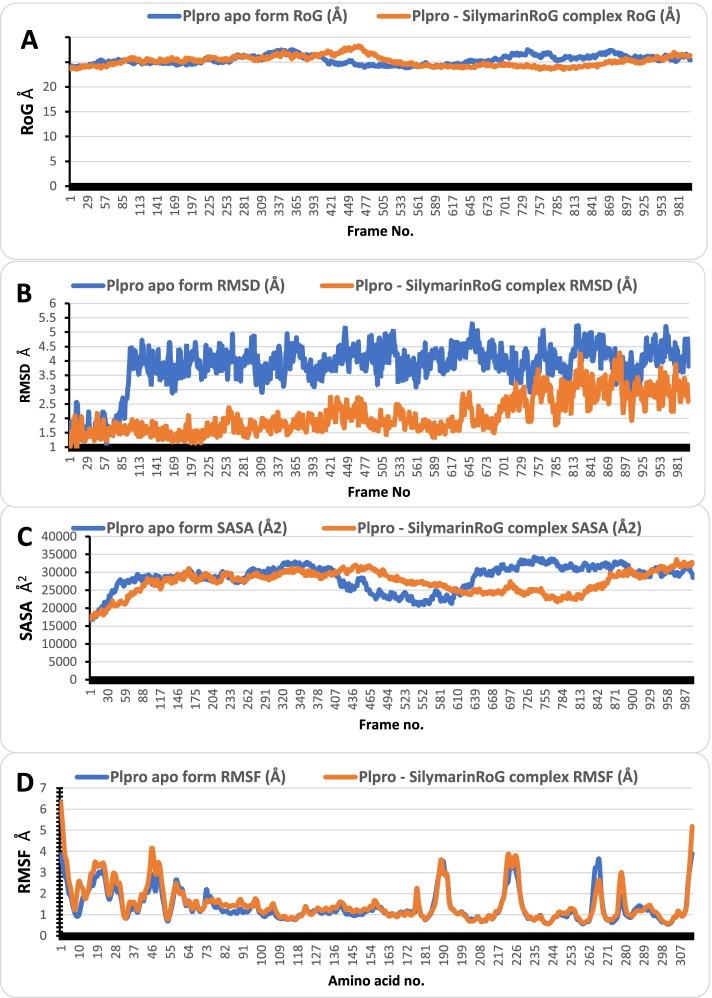
The SASA, RoG, RMSD, and RMSF of PLpro apo form (blue) and PLpro-Silymarin complex (orange)

**Fig. 5:**
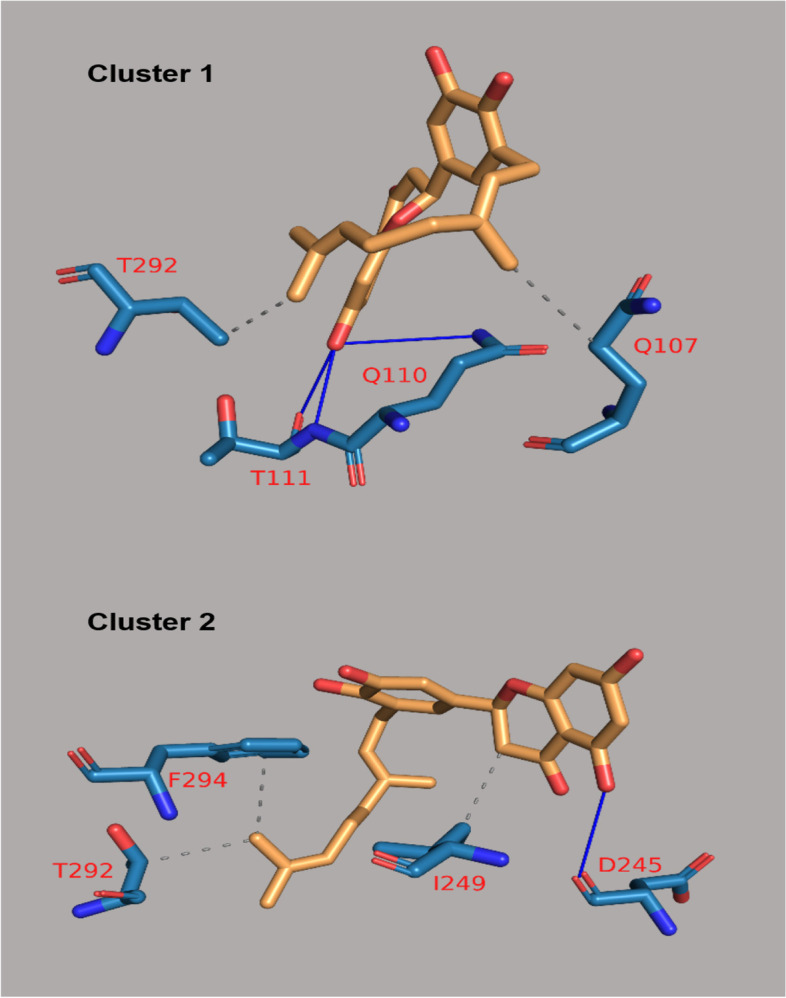
Types of Interaction between Isonymphaeol B and 3CLpro in the cluster representatives. Gray dashed lines are hydrophobic interactions. Blue solid lines are hydrogen bonds. Protein residues are in blue stick representative with their one-letter code in red. Isonymphaeol B in orange stick representative

**Fig. 6:**
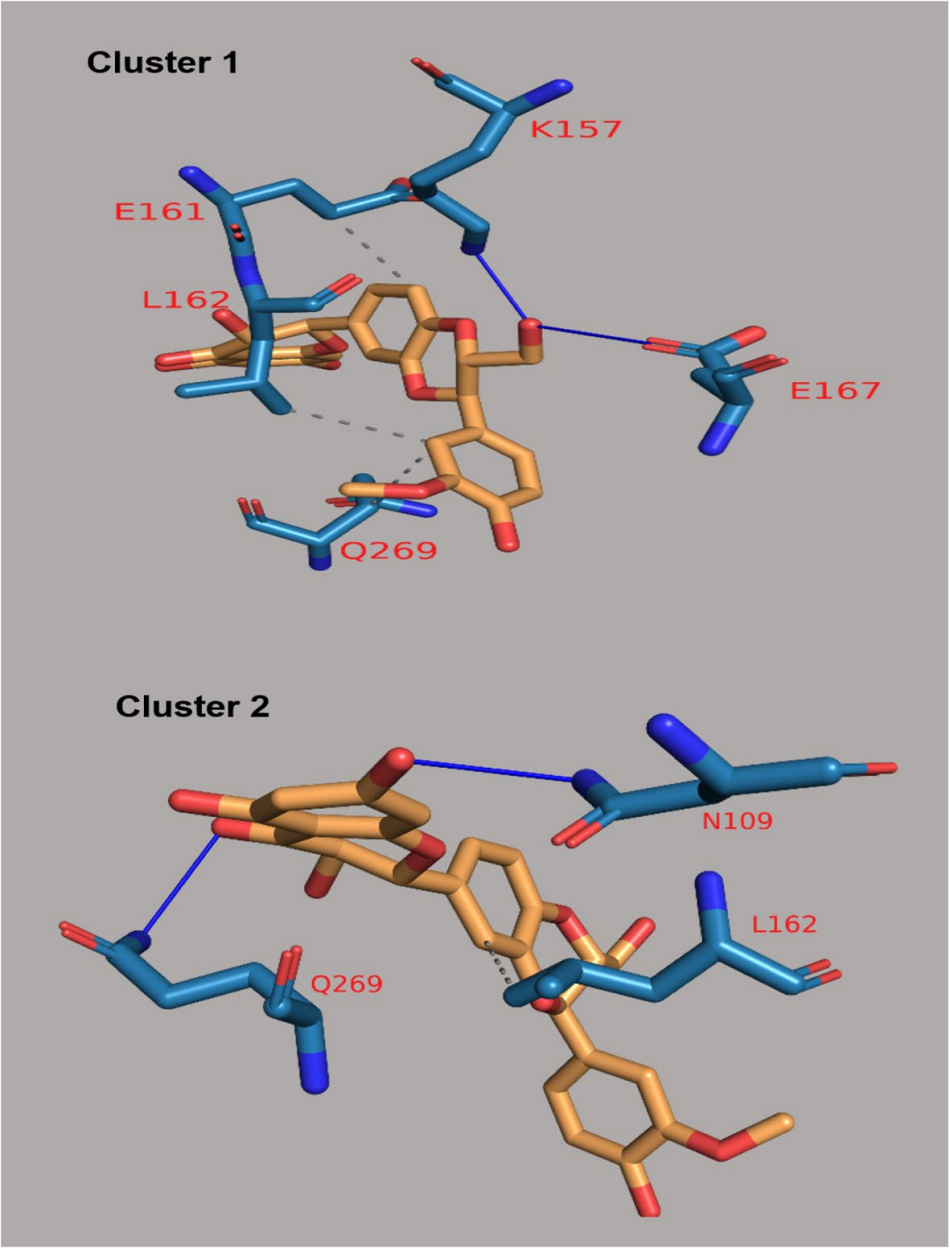
Types of Interaction between Silymarin and PLpro in the cluster representatives. Gray dashed lines are hydrophobic interactions. Blue solid lines are hydrogen bonds. Protein residues are in blue stick representative with their one-letter code in red. Silymarin is in orange stick representative

The MDS was performed on 3CLpro and PLpro in apo form and in complex with isonymphaeol B and silymarin, respectively for 100 ns in NVT ensemble. After that, SASA, RMSD, RoG, and RMSF were calculated (Figs. 3 and 4). For 3CLpro apo form, the average values of SASA, RMSD, RoG, and RMSF were 27800Å2, 1.76 Å, 25.29 Å, and 1.06 Å, respectively, while its complex with isonymphaeol B fluctuated around 27779Å2, 1.81 Å, 25.74 Å, and 1.04 Å, respectively. On the other hand, PLpro apo form had average values of SASA, RMSD, RoG, and RMSF as 28516Å2, 3.781 Å, 25.42 Å, and 1.38 Å, respectively and its complex with silymarin had average values of 27,188 Å2, 2.1 Å, 25.18 Å, and 1.52 Å, respectively. The RMSF values had spikes at the start and the end of the protein, which corresponded to the fast motion of the terminals while the spikes in the middle corresponded to the motion of the loops. The lower average RSMD value for the complexes indicates that the binding of the flavonoids increased the stability of the proteins. The number of clusters produced from TTClust, the types and number of interactions from PLIP webserver are presented in Table 6, while the interactions between the compounds and protein images are represented in Figs. 5 and 6.

**Table 6:** Clusters and interactions of 3CLpro-Isonymphaeol B and PLpro-Silymarin

Protein- Compound complex	Cluster number	Hydrophobic interactions		Hydrogen bonds	
		Number of interactions	Amino acids involved in the interaction	Number of interactions	Amino acids involved in the interaction
3CLpro-Isonymphaeol B	Cluster 1	2	Q107 – **T292**	3	Q110 – T111 (2)
	Cluster 2	3	I249 – **T292**—F294	1	D245
PLpro-Silymarin	Cluster 1	3	G161 – **D162**—G269	2	V157 – M167
	Cluster 2	1	**D162**	2	C109 – G269

## Discussion

Therapeutic compounds against COVID-19 could stimulate human cells such as receptors and immune system. Also, structural and non-structural proteins of virus could be targeted by compounds. Compounds that target the virus inhibit binding of virus to human cell receptors, prevents viral replication processes, inhibit viral protein modification and/or inhibit self-assembly process of the virus. Viral spike protein, 3CLpro, PLpro, RNA-dependent RNA polymerase, and host cell proteins (ACE2 and TMPRSS2) that facilitate viral entry to cell are important therapeutic targets.

Cell entry by virus is followed by translation of the two-thirds of the genome, from the 5' end to yield two large replicase polyproteins (pp1a and pp1ab), which are cleaved by PLpro and 3CLpro to yield up to 16 non-structural proteins (nsp1 – nsp16). The assembly of the proteins give rise to membrane-bound replicase complex, which facilitate replication and structural gene translation of viral genome [7, 48]. In addition to the N-terminus proteolytic cleavage activities of PLpro to yield nsp1, nsp2 and nsp3, the protease also antagonizes the host’s innate immunity via its deubiquitinating/deISGylating activity [49–51]. Further downstream, proteolytic cleavage of polyproteins by 3CLpro yields nsp4 –nsp16 [52].

The amino acid residues Cys111, His272 and Asp286 forms the catalytic triad of SARS-CoV-2 PLpro [7, 48], while Trp106, Gly256, and Lys274 are amino acid residues at the catalytic region [48]. Also, residues Glu167, Leu162, Asp164 and Tyr264 have been reported to be essential or required for deubiquitinating activity of PLpro [53]. The response of host innate immune system is critical to controlling SARS-CoV-2 infection. The PLpro of SARS-CoV-2 reverses post-translational modification of immune proteins, like interferon factor 3 and NF-κB, which is achieved by ubiquitin and interferon-stimulated gene product 15 (ISG15). The reversal of ubiquitination and ISGylation suppresses host innate immune responses [7, 54, 55], and assists SARS-CoV-2 escape from host innate immune responses. The results revealed that licorice, the flavonoid with the lowest binding energy, interacted with HIS89, ASN109B, GLY160A, ASN109C, GLY160C HIS89, VAL159, GLY160, and did not interact with amino acids required for post-translational and deubiquitination activities of PLpro, thus it may not directly inhibit these activities. However, licorice, through its strong interactions with neighbouring amino acid residues may cause some conformational changes at the active site which may indirectly inhibit PLpro. However, lopinavir (a reference protease inhibitor used in this study) interacted with Leu162 of PLpro via Pi-alkyl interaction, suggesting that it may inhibit the deubiquitinating activity of PLpro. The interaction of procyanidin with PLpro, via its Pi-Sigma interaction with Trp106, Pi-alkyl interaction with Leu162 and Pi–Pi interaction with Tyr264 (amino acids required for the deubiquitinating activity of the enzyme), suggests that procyanidin may alter the catalytic conformation of PLpro and inhibit its ability to reverse ubiquitination. The interactions of silymarin via Pi-donor hydrogen bond, ugonin M via alkyl interaction, gallocatechin gallate and isonymphaeol B via Pi-alkyl interaction with Leu162 of PLpro suggests that the flavonoids may possess inhibitory activities against the deubiquitinating function of SARS-CoV-2 PLpro, thereby limiting the ability of the virus to suppress the host immunity. The results suggest that procyanidin may be more potent in inhibiting the deubiquitinating activity of SARS-CoV-2 PLpro compared to other flavonoids and lopinavir (the reference compound).

The catalytic dyad (His41 and Cys145) of 3CLpro is domiciled between its domain I (residues 8–101) and domain II (residues 102–184) [56]. A long loop (residues 185–200) that connects domain II and domain III (residues 201–303) completes the 3CLpro monomer [52]. The 3CLpro recognises Leu-Gln*Ser, Leu-Gln*Ala and Leu-Gln*Gly sequence at most sites for cleavage (cleavage site asterisked) [56]. 3CLpro is a good therapeutic target as no human protease appears to have such recognition sequence [56]. The interaction of lopinavir and ritonavir with Gln110 of 3CLpro occurred via hydrogen bonding while baicalin and apigetrin interacted with the amino acid residue via a Pi-donor hydrogen bond. Both isonymphaeol B and abyssinone II interacted with a number of the same amino acids of 3CLpro (Asp295, Phe294, Pro293, Ile249, Val202, and His246) as did lopinavir. In addition to these, isonymphaeol B also interacted with Asp153 while abyssinone II interacted with Ile200 (a residue that ritonavir also interacted with), both amino acids being points of interaction for lopinavir. While lopinavir interacted with ASP295, a domain III amino acid residue of 3CLpro, via a Pi-anion bond, isonymphaeol B and abyssinone II interacted via hydrogen bonding. The large number of hydrogen bonds involved in the interaction of flavonoids with the least binding energies (isonymphaeol B, baicalin, abyssinone II, apigetrin, and tomentin A) with 3CLpro seems to be responsible for the higher binding affinities of the protein for them. Despite that these flavonoids did not interact with the catalytic dyad of 3CLpro, their strong interactions with neighbouring amino acid residues may cause some conformational changes at the active site which may inhibit the catalytic activity of 3CLpro. The strong hydrogen bonding and hydrophobic interaction exhibited by isonymphaeol B suggest its potential as a potent 3CLpro inhibitor. The anti-SARS-CoV-2 activities of baicalin and silymarin, have similarly being reported by Akhter et al. [29]. The flavonoids were identified as potentially active compounds against SARS-CoV-2 Mpro.

The predictive physicochemical and pharmacokinetic analyses of flavonoids with the least binding energies revealed variations in the properties of these compounds. The results revealed that the gastrointestinal absorption was high for isonymphaeol B, abyssinone II and tomentin A but was low for other flavonoids with low binding energies for PLpro and 3CLpro (FLBEPCs), suggesting that the bioavailability of these three compounds is high compared to others. Permeability glycoprotein (P-gp) is extensively expressed in the intestinal epithelium, liver cells, proximal tubular cells of the kidney and capillary endothelial cells comprising the blood–brain barrier and blood-testis barrier, where it pumps xenobiotics back into the intestinal lumen, bile ducts, urine-conducting ducts and capillaries respectively [57]. From the results, licorice, ugonin M, baicalin, apigetrin and tomentin A were substrates for P-gp, suggesting that their absorption into the earlier mentioned tissues will be low, thereby affecting their bioavailability and increasing their excretion, which in turn, will shorten their half-lives. SARS-CoV-2 has been reported to infect the brain, thus indicating its ability to cross the blood brain barrier (BBB) [58]. Of all the FLBEPCs, only abyssinone II has the potential of crossing the BBB. Thus, it may be able to clear the viral load in the brain. Lipinski’s rule of five has always been used to evaluate the drug-likeness of any compound; the more the violation of the rule, the less the drug-likeness of the compound [40]. Of all the FLBEPCs, only isonymphaeol B, ugonin M, silymarin, abyssinone II, apigetrin and tomentin A did not violate any of the five rules, thus suggesting that they had higher drug-likeness compared to others.

The toxicities of the FLBEPCs were also evaluated in silico. hERG channel plays a vital role in the repolarization and termination stages of action potential in cardiac cells [59, 60]. hERG channel blockers cause cardiotoxicity [61]. The potentials of these compounds as hERG channel blockers were evaluated. The results revealed that only ugonin M, isonymphaeol B, apigetrin and tomentin A did not exhibit the potential of being hERG channel blockers, suggesting that they may not cause hERG channel-related cardiotoxicity. Also, the mutagenicities of the FLBEPCs were evaluated in silico. The results revealed that gallocatechin gallate, baicalin and apigetrin exhibited mutagenicity in silico. Thus, they are probable mutagens, which can cause genetic mutations, which, in turn, may initiate the pathophysiology of other diseases, such as cancer. The liver is exposed to higher concentration of drugs, being the primary organ responsible for drug metabolism. In this study the effects of the compounds on the liver were evaluated. The results indicated that only ugonin M, abyssinone II and apigetrin were hepatotoxic. Of all the FLBEPCs, only ugonin M, silymarin, isonymphaeol B and abyssinone II exhibited the potential to be inhibitors of different variants of cytochrome P450, thus they may adversely affect phase I drug metabolism in the liver. Thus, of all the flavonoids studied, isonymphaeol B may be predicted as the most effective inhibitor of 3CLpro with favourable pharmacokinetic parameters and no toxicity while procyanidin may be predicted as the most effective inhibitor of PLpro with less favourable pharmacokinetic parameters, drug-likeness and low toxicity. However, the low drugability of procyanidin (violating 3 out of the Lipinski’s rule of five) suggests that further chemical modification of the structure of the compound is required in order to increase this parameter. Based on this, silymarin may be a better alternative, though it is more toxic than procyanidin.

The MDS was performed on 3CLpro and PLpro in apo form and in complex with isonymphaeol B and silymarin, respectively for 100 ns in NVT ensemble. Furthermore, the SASA, RMSD, RoG, and RMSF were calculated from the trajectory of MD simulation. The RMSD parameter demonstrate insight into the structural conformations of proteins through molecular dynamics simulation [62]. Through this parameters, the stability of protein backbone can be analysed when bound with a ligand or small molecule. Low values during RMSD runs indicates high stability of protein ligand system, while high values during RMSD refers to comparatively low stability of the system [62]. While lower RMSD values are considered ideally acceptable for protein systems [63]. The 3CLpro-Isonymphaeol B and PLpro-Silymarin complexes demonstrated appreciable degree of stability throughout the period of the 100 ns MDS run. For each of the representative conformers for the selected clusters from the clustering analysis, it was observed that the interactions were maintained at different time frames compared to the initial interactions, indicating that the interaction can be maintained in a dynamic environment, thus can be well adapted for experimental procedures.

## Conclusion

This study evaluated the inhibitory potentials of selected flavonoids against PLpro and 3CLpro of SARS-CoV-2. The results revealed that silymarin and isonymphaeol B exhibited better binding energies, demonstrated appreciable degree of stability during MDS run, complied with Lipinski rule for drug-likeness and exhibited favourable pharmacokinetics in silico suggesting them as potential inhibitors of PLpro and 3CLpro. Thus, they are candidates that may be considered for further studies as potential COVID-19 therapeutic agents.

## Supplementary Information

**Additional file 1.**

## Data Availability

Supplementary Data are available and attached.

## Abbreviations

3CLpro: 3-Chymotrypsin-like protease
COVID-19: Coronavirus disease 2019
FLBEPCs: Flavonoids with low binding energies for PLpro and 3CLpro
MERS-CoV: Middle East respiratory syndrome coronavirus
PLpro: Papain-like protease
SARS: Severe acute respiratory syndrome
SARS-CoV: Severe acute respiratory syndrome coronavirus
SARS-CoV-2: Severe acute respiratory syndrome coronavirus-2
