# Supplementary material for: Pharmacoinformatic study of inhibitory potentials of selected flavonoids against papain-like protease and 3-chymotrypsin-like protease of SARS-CoV-2
Source: Clin Phytosci. 2022 Sep 8;8(1):16. doi: 10.1186/s40816-022-00347-y (PMC9452863; doi:10.1186/s40816-022-00347-y)
Supplement: Supplementary file 1 — Additional file 1. [file 40816_2022_347_MOESM1_ESM.doc]

**SUPPLEMENTARY MATERIAL**

**Pharmacoinformatic Study of Inhibitory Potentials of** **Selected Flavonoids against Papain-Like Protease and 3-Chymotrypsin-Like Protease of SARS-CoV-2**

Table S1 Binding Energies and Amino Acid Interactions of Flavonoids with Papain-like Protease (PLpro) of SARS-CoV-2

| **S/N** | **Compound**  **(PubChem CID)** | **Chemical structure** | **2D diagram of ligand interaction with amino acids** | **Hydrogen bonding–related residues** | **Non- hydrogen bonding–related residues** | **Binding Energy (Kcal/mol)** |
| --- | --- | --- | --- | --- | --- | --- |
| R1 | Lopinavir*  (92727) | 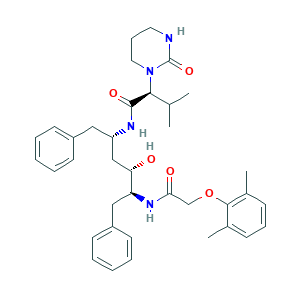 | 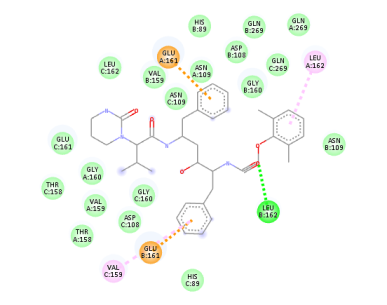 | LEU162 | GLU161B, VAL159, GLU161A, LEU162 | -9.1 |
| R2 | Ritonavir*  (392622) | 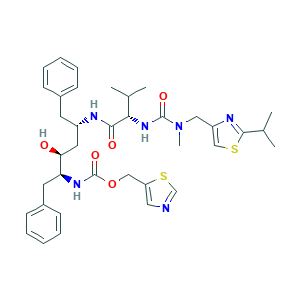 | 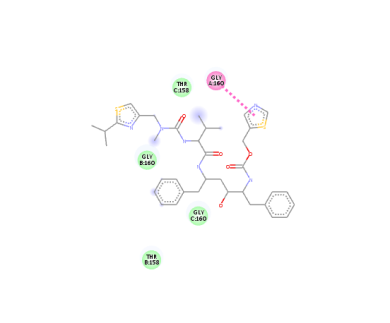 | ………. | GLY160 | -8.5 |
| 1 | Licorice  (163463) | 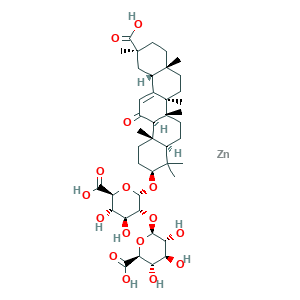 | 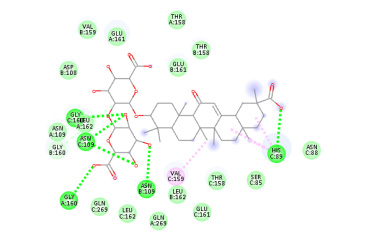 | HIS89, ASN109B, GLY160A, ASN109C, GLY160C | HIS89, VAL159, GLY160 | -11.8 |
| 2 | Ugonin M  (135891244) | 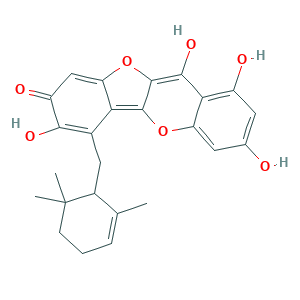 | 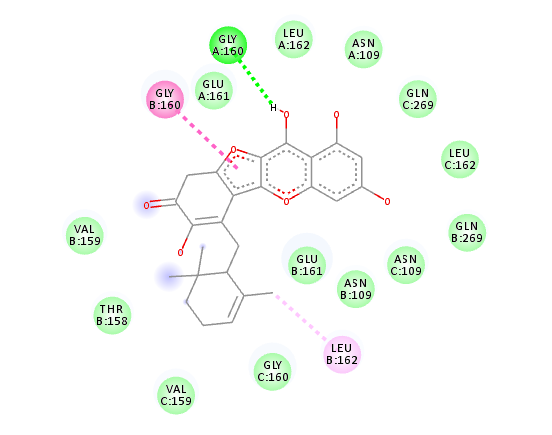 | GLY160A | LEU162B, GLY160B | -10.1 |
| 3 | Procyanidin  (107876) | 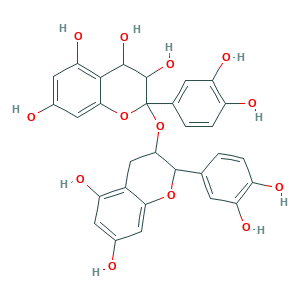 | 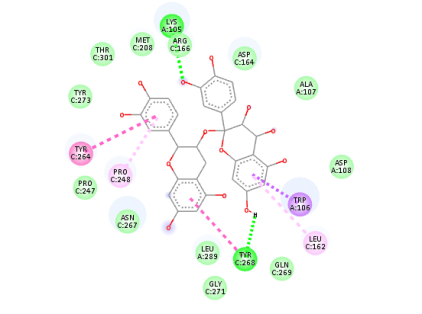 | TYR268, LYS105 | TYR268, LEU162, TRP106, PRO248, TYR264 | -9.8 |
| 4 | Silymarin  (5213) | 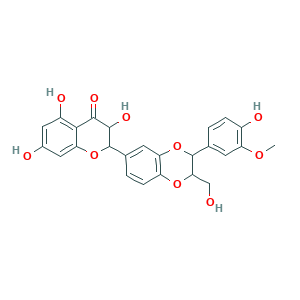 | 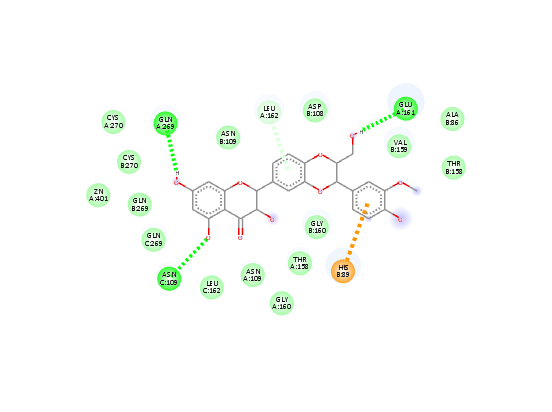 | ASN108C, GLN2691, GLU161A | HIS29B, LEU162A | -9.7 |
| 5 | Gallocatechin gallate  (5276890) | 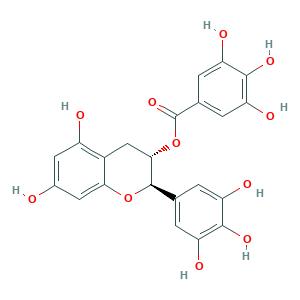 | 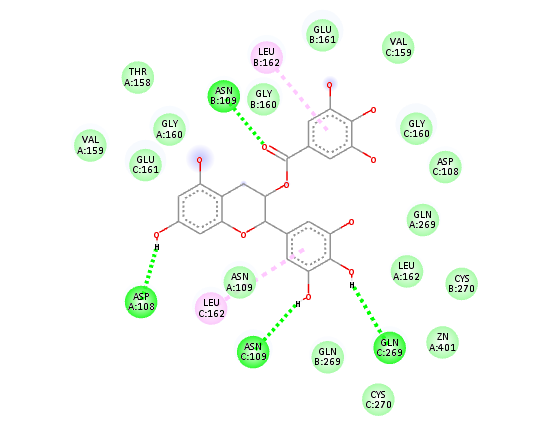 | ASN109B, ASP108, GLN269, ASN109C | LEU162B, LEU162C | -9.6 |
| 6 | Epigallocatechin gallate  (65064) | 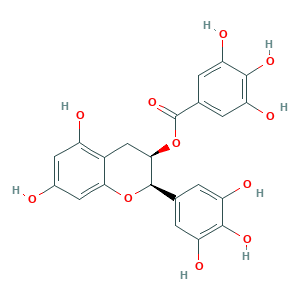 | 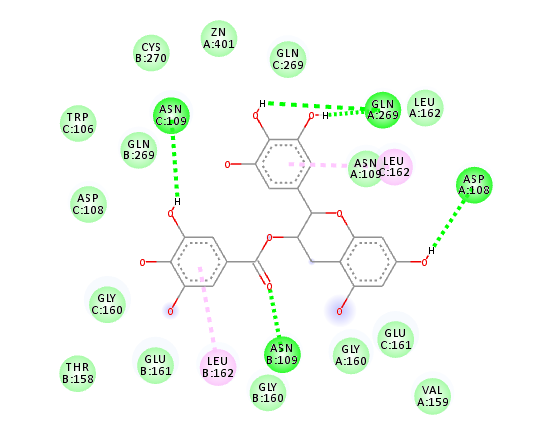 | ASN109C, GLN269, ASP108, ASN109B | LEU162C, LEU162B | -9.6 |
| 7 | Isoquercetin  (5280804) | 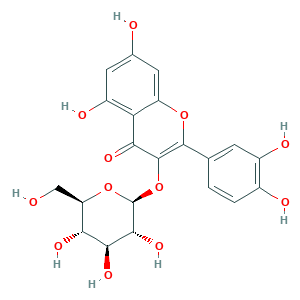 | 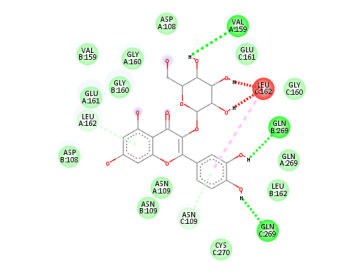 | VAL159, GLN269, GLN269 | LEU162C, ASN109, LEU162A | -9.3 |
| 8 | Isonymphaeol B  (10070991) | 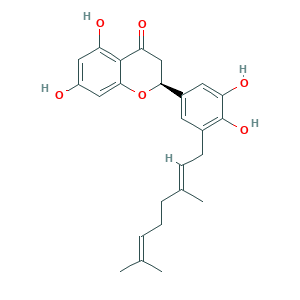 | 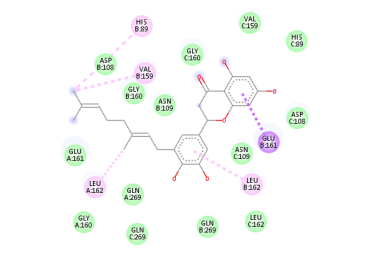 | ….. | GLU161B, LEU162B, LEU162A, VAL159B, HIS89B | -9.3 |
| 9 | Baicalin  (64982) | 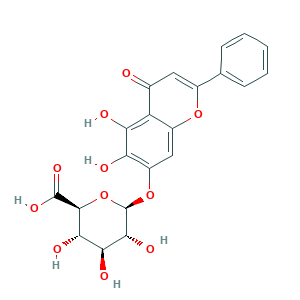 | 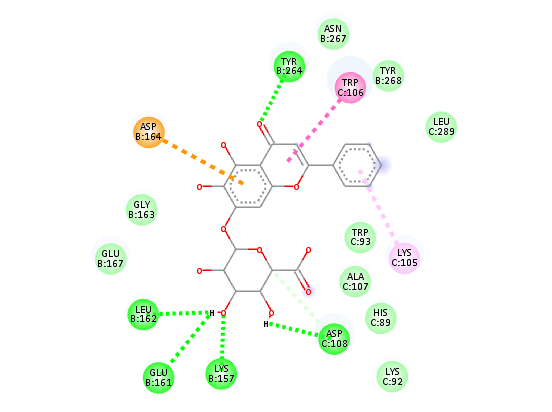 | ASP108, LYS157, GLU161, LEU162, TYR264 | TRP106, LYS105, ASP164, ASP108 | -9.2 |
| 10 | Hyperin  (5281643) | 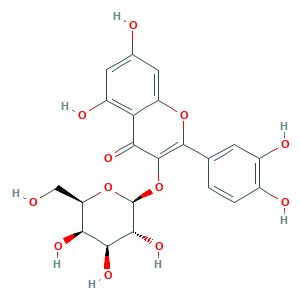 | 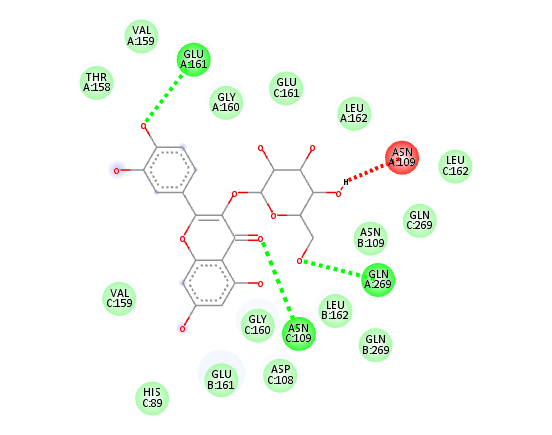 | GLU161, ASN109, GLN269 | ASN109 | -9.1 |
| 11 | Flavopiridol  (5287969) | 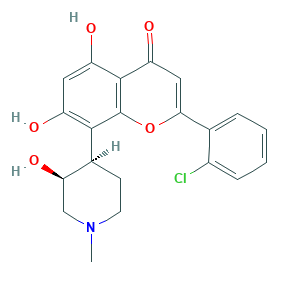 | 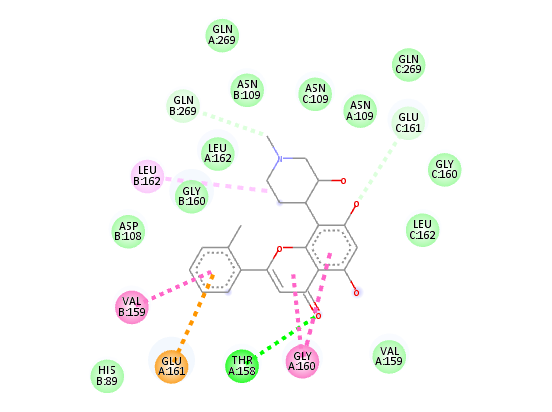 | THR158 | GLY160, GLU161A, VAL159, LEU162, GLN269, GLU161C | -9.0 |
| 12 | Apigetrin  (5280704) | 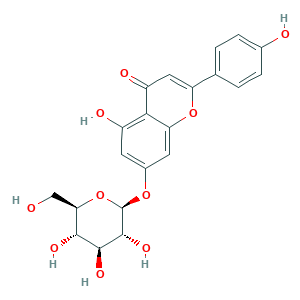 | 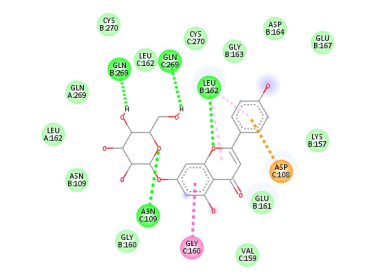 | ASN109, GLN269, GLN269, LEU162 | ASP108, LEU162 | -8.9 |
| 13 | Houttuynoid B  (57412150) | 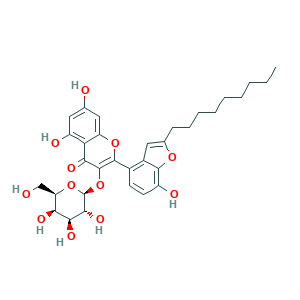 | 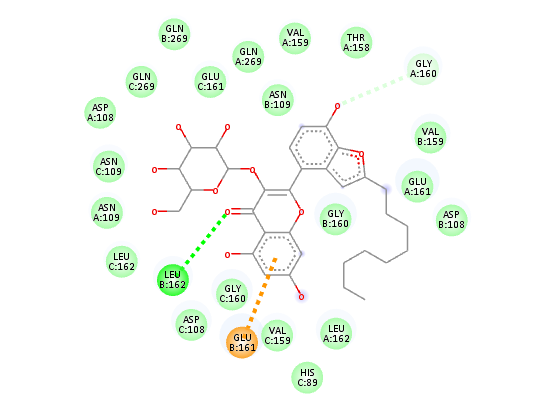 | LEU162 | GLU161, GLY160 | -8.9 |
| 14 | Methoxymatteucin  (158031) | 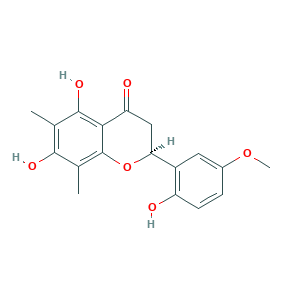 | 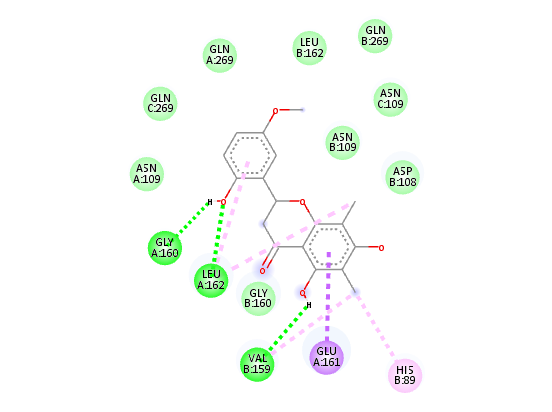 | VAL159, LEU162, GLY160 | LEU162, VAL159, GLU161, HIS89 | -8.8 |
| 15 | Rutin  (5280805) | 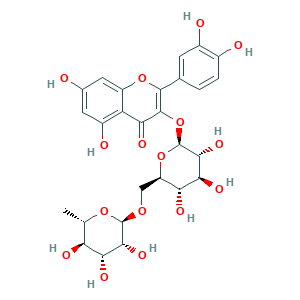 | 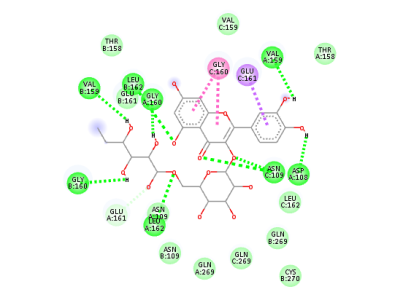 | VAL159A, ASP108A, ASN109C, LEU162A, GLY160B, VAL159B, LEU162B, GLY160A | GLU161A, GLY160C, GLU161C | -8.8 |
| 16 | Astragalin  (5282102) | 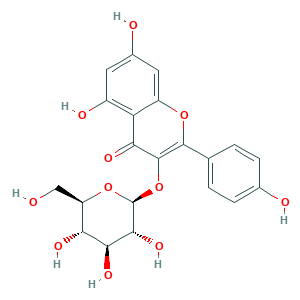 | 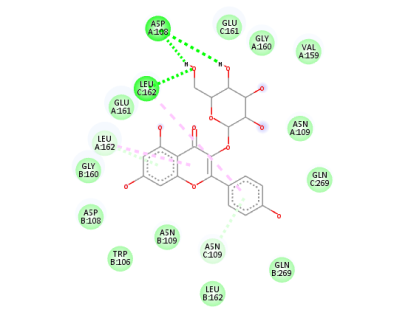 | ASP108, LEU162 | LEU162C, LEU162A, ASN109 | -8.7 |
| 17 | Kaempferol-3-O-glucorhamnoside  (5318761) | 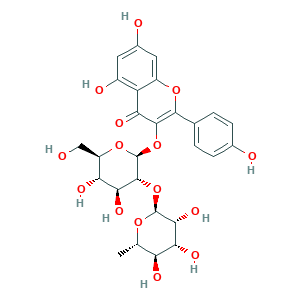 | 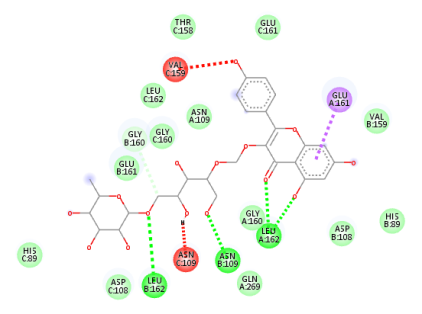 | LEU162A, LEU162B, ASN109 | ASN109, VAL159, GLY160, GLU161 | -8.7 |
| 18 | Tomentin A  (71659627) | 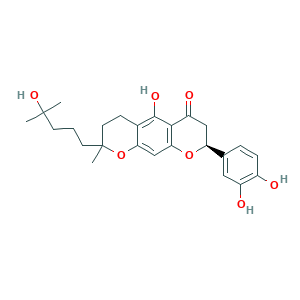 | 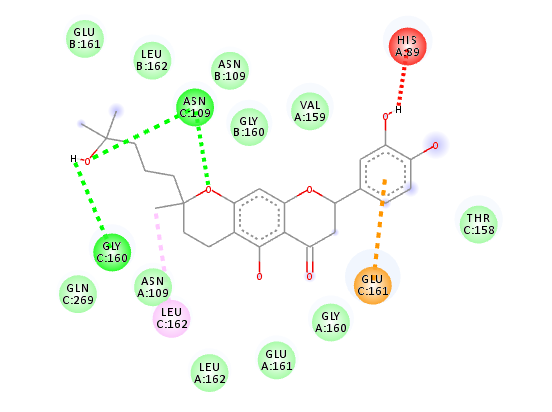 | ASN109C, GLY160C | HIS89A, GLU161C, LEU162C | -8.7 |
| 19 | Tomentin B  (71659628) | 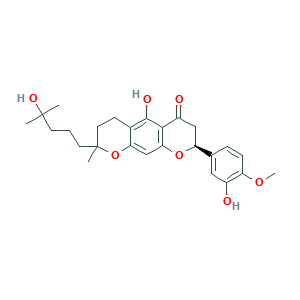 | 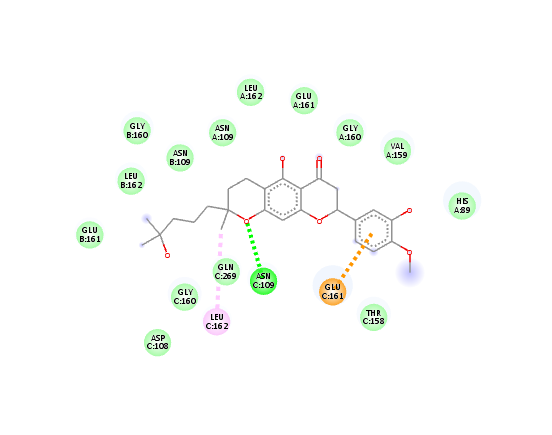 | ASN109C | GLU161, LEU162 | -8.7 |
| 20 | Abyssinone II  (10064832) | 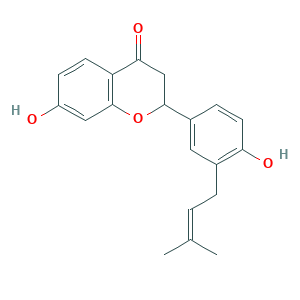 | 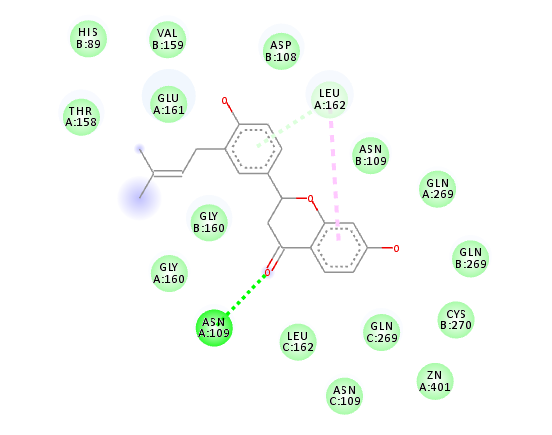 | ASN109 | LEU162 | -8.6 |
| 21 | Rhoifolin  (5282150) | 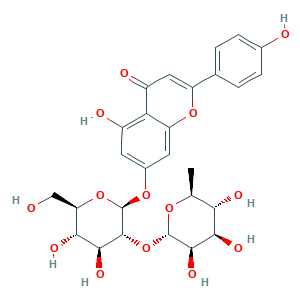 | 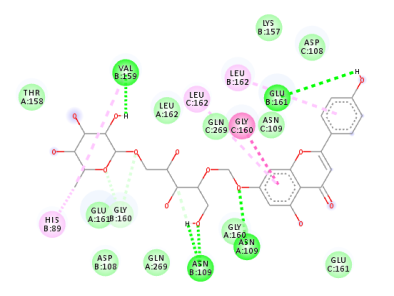 | GLU161, ASN109A, ASN109B, VAL159 | GLY160, ASN109, VAL159, LEU162, GLY160, LEU162 | -8.6 |
| 22 | Sanggenol A  (15233693) | 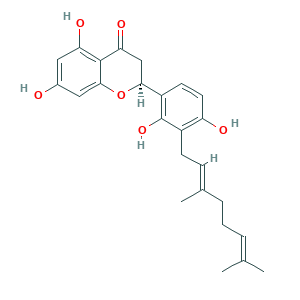 | 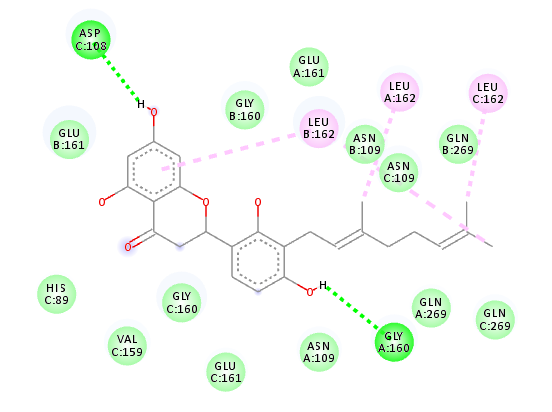 | GLY160A, ASP108C | LEU162B, LEU162A, LEU162C | -8.6 |
| 23 | Liquiritin apioside  (10076238) | 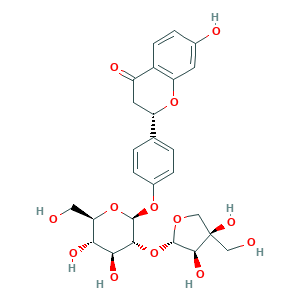 | 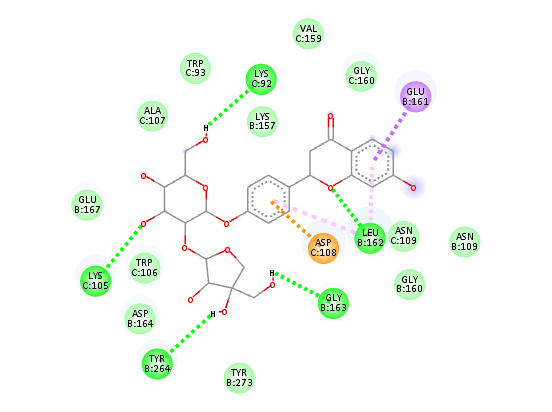 | LEU162, GLY163, TYR264, LYS105, LYS92 | GLU161, LEU162, ASP108 | -8.5 |
| 24 | Tomentin E  (71659767) | 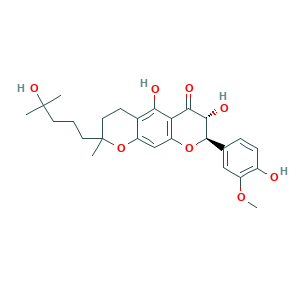 | 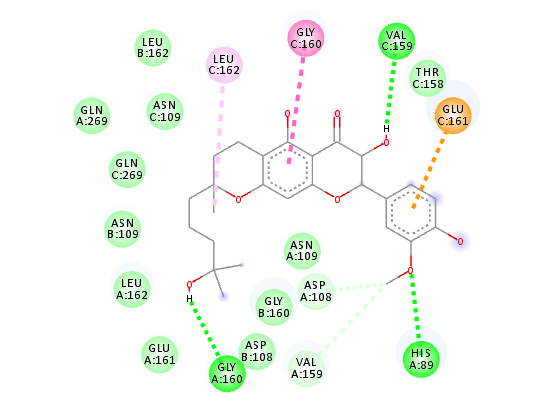 | VAL159C, HIS89A, GLY160A | VAL159A, ASP108A, LEU162C, GLY160C, GLU161C | -8.5 |
| 25 | Fisetin  (5281614) | 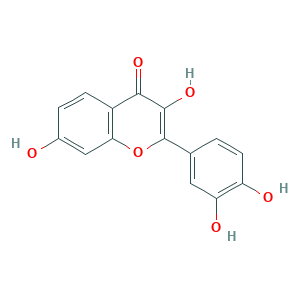 | 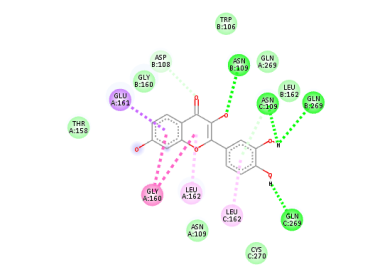 | ASN109B, ASN109C, GLN269B, GLN269C | ASP108, GLU161, GLY160, LEU162A, LEU162C, ASN109 | -8.4 |
| 26 | Lophirone A  (5488805) | 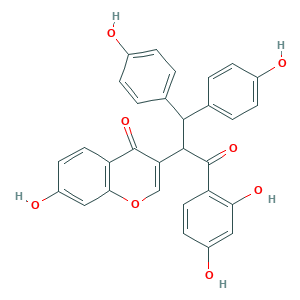 | 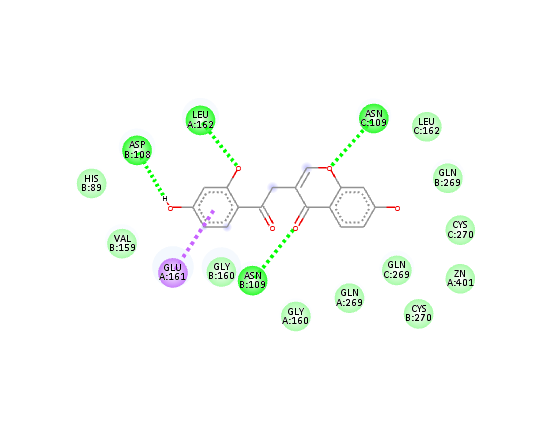 | ASN109B, ASP108, LEU162, ASN109C | GLU161 | -8.4 |
| 27 | Quercetin  (5280343) | 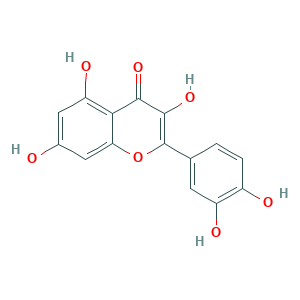 | 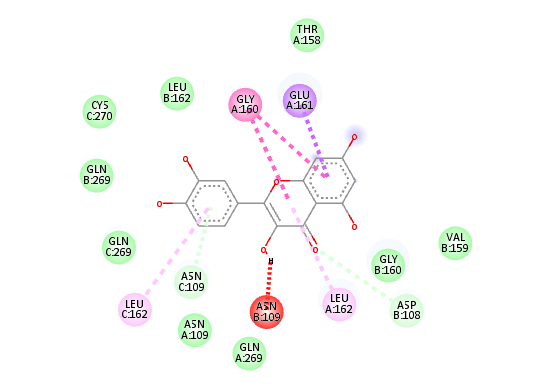 | …………. | ASP108, LEU162, ASN109B, ASN109C, LEU162, GLY160, GLU161 | -8.4 |
| 28 | Quercitrin  (5280459) | 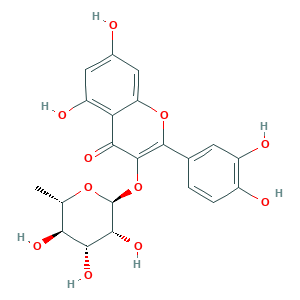 | 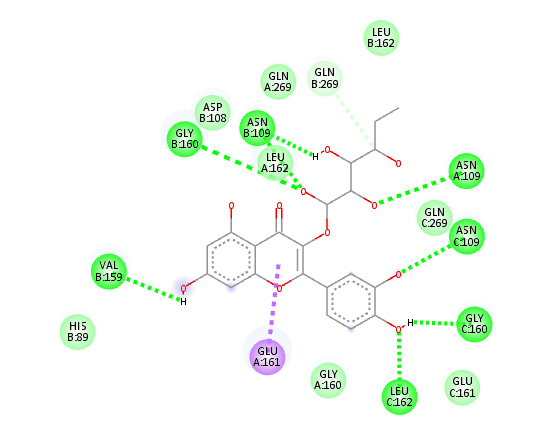 | ASN109A, ASN109C, GLY160C, LEU162, VAL159, GLY160B, ASN109B | GLN269, GLU161 | -8.4 |
| 29 | 3,4'-dihydroxyflavone  (145726) | 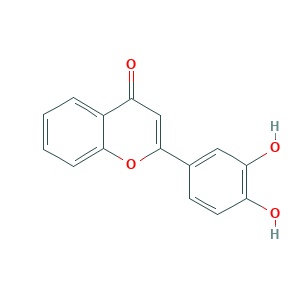 | 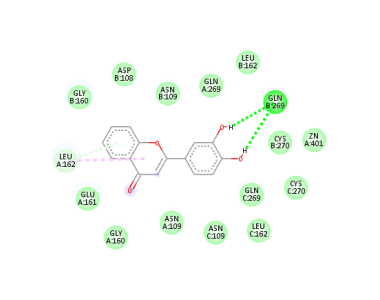 | GLN269 | LEU162 | -8.3 |
| 30 | Baicalein  (5281605) | 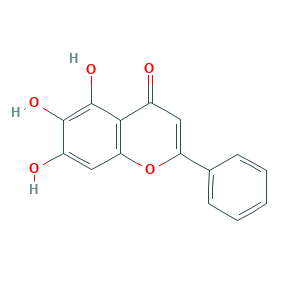 | 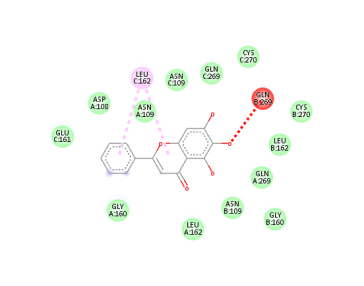 | ……….. | LEU162, GLN269 | -8.3 |
| 31 | Isorhamnetin  (5281654) | 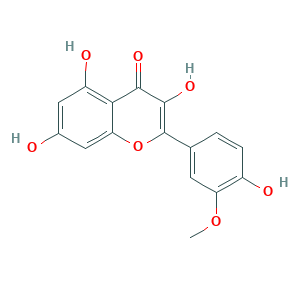 | 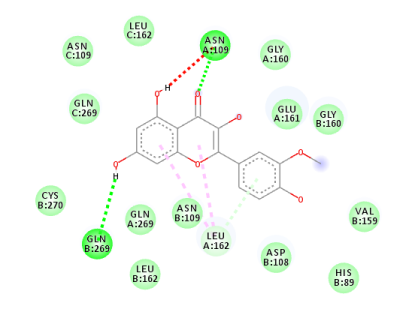 | GLN269, ASN109 | ASN109, LEU162 | -8.3 |
| 32 | Dihydroquercetin  (439533) | 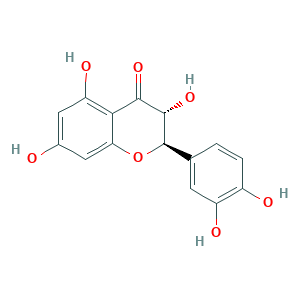 | 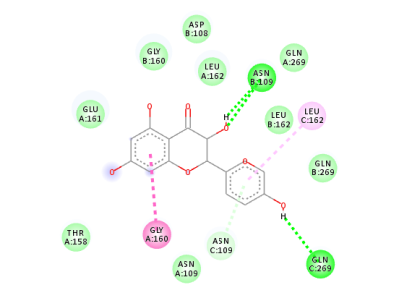 | ASN109, GLN269 | LEU162, ASN109, GLY160 | -8.2 |
| 33 | Kaempferide  (5281666) | 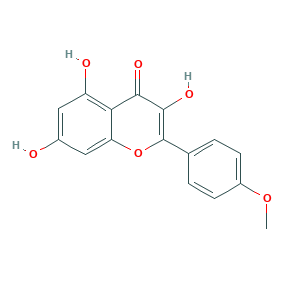 | 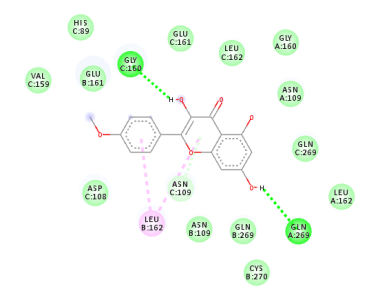 | GLN269, GLY160 | LEU162, ASN109 | -8.2 |
| 34 | Agathisflavone  (5281599) | 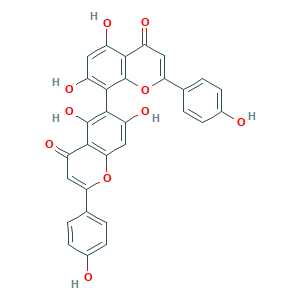 | 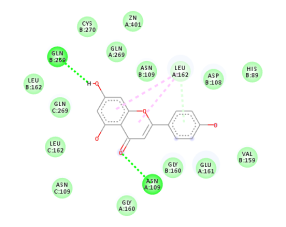 | GLN269, ASN109 | LEU162 | -8.1 |
| 35 | Bedaquiline  (5388906) | 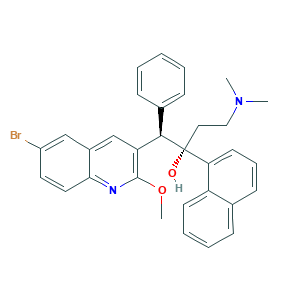 | 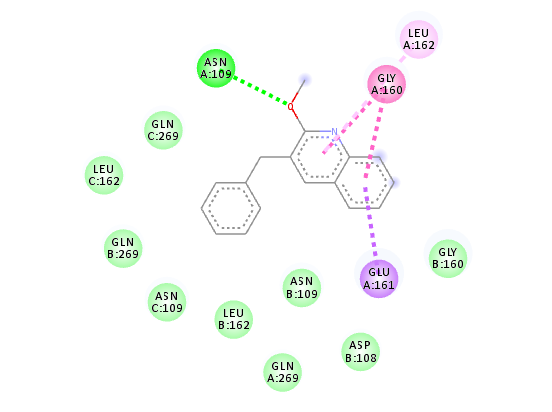 | ASN109 | GLU161, GLY160, LEU162 | -8.1 |
| 36 | Diosmetin  (5281612) | 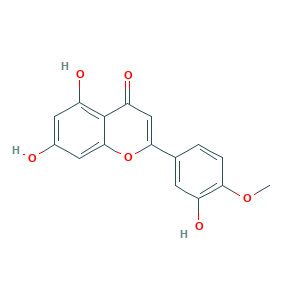 | 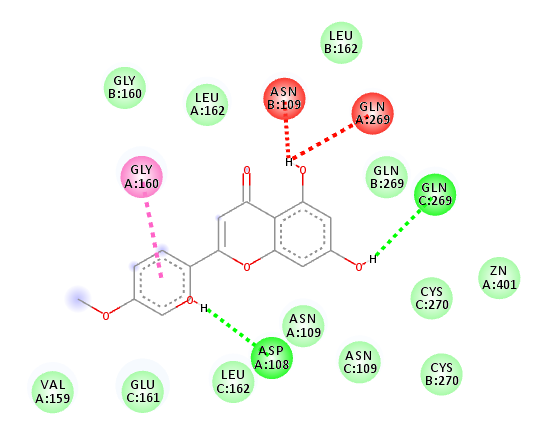 | GLN269, ASP108 | ASN109, GLN269, GLY160 | -8.1 |
| 37 | Catechin  (9064) | 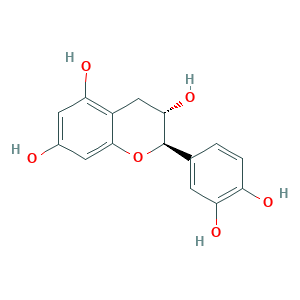 | 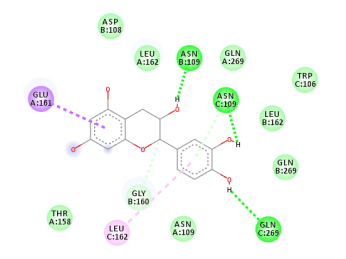 | ASN109B, ASN109C, GLN269 | LEU162, GLU161, GLY160, ASN109 | -8.0 |
| 38 | Galangin  (5281616) | 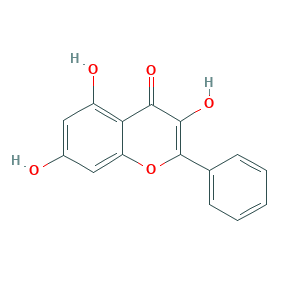 | 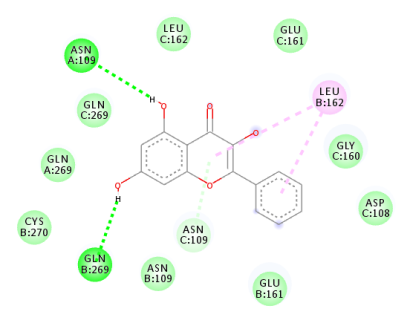 | ASN109, GLN269 | LEU162, ASN109 | -8.0 |
| 39 | Herbacetin  (5280544) | 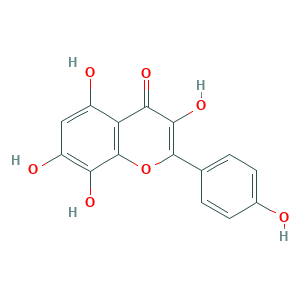 | 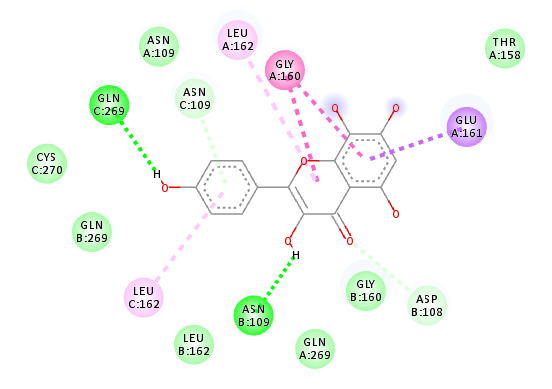 | GLN269, ASN109 | ASP108, LEU162C, ASN109, LEU162A, GLY160, GLU161 | -8.0 |
| 40 | Jaceosidin  (5379096) | 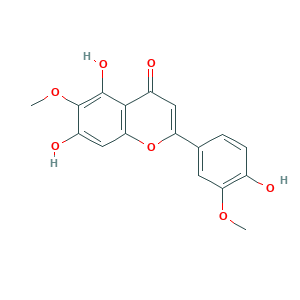 | 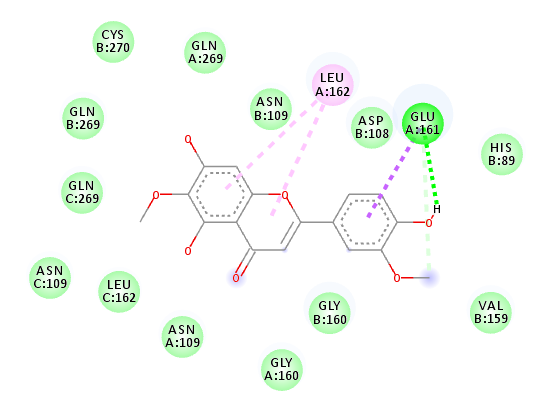 | GLU161 | GLU161, LEU162 | -8.0 |
| 41 | Myricetin  (5281672) | 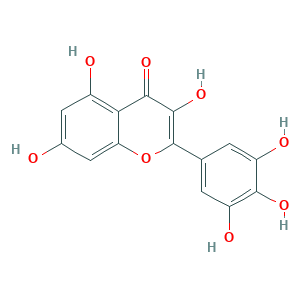 | 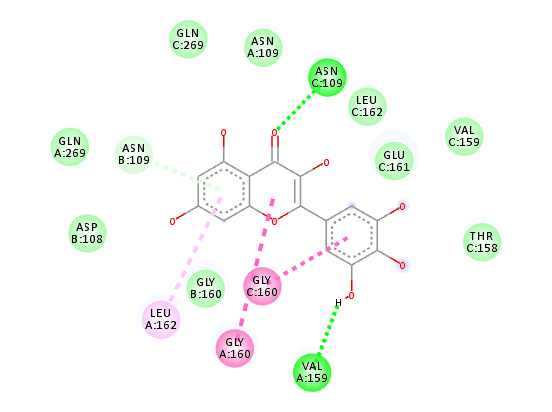 | VAL159, ASN109 | GLY160A, GLY160C, LEU162, ASN109 | -8.0 |
| 42 | Naringenin  (932) | 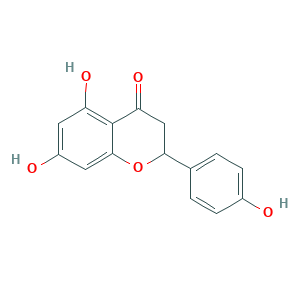 | 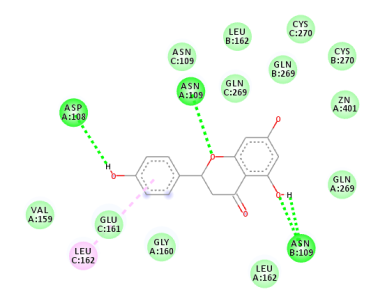 | ASN109A, ASP108, ASN109B | LEU162 | -8.0 |
| 43 | Chrysin  (5281607) | 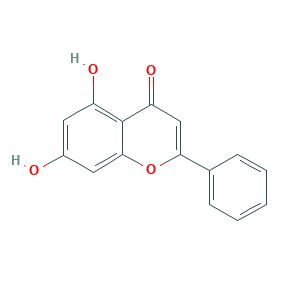 | 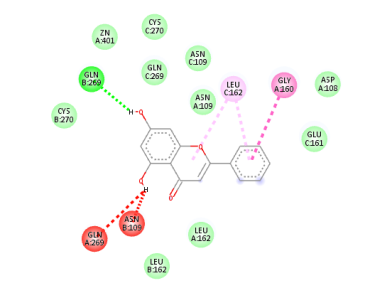 | GLN269 | LEU162, GLY160, GLN269, ASN109 | -7.9 |
| 44 | Chrysosplenol C  (189065) | 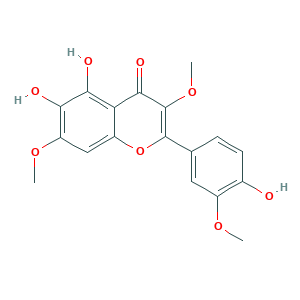 | 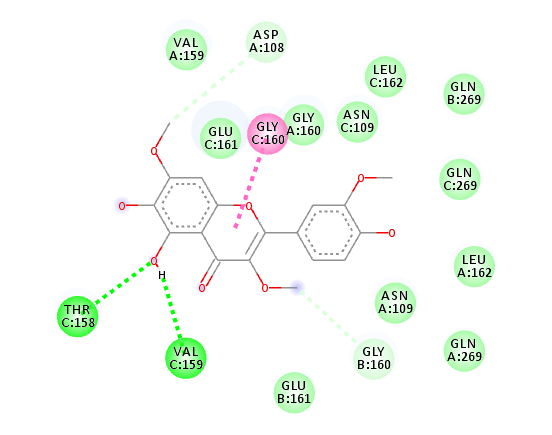 | THR158, VAL159 | ASP108, GLY160, GLY160 | -7.9 |
| 45 | Isobavachalcone  (5281255) | 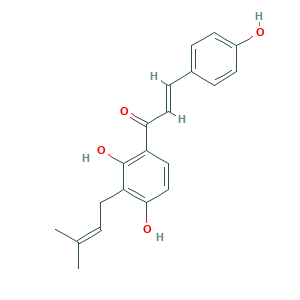 | 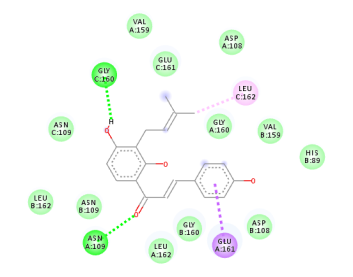 | GLY160, ASN109 | GLU161, LEU162 | -7.9 |
| 46 | Norwogonin  (5281674) | 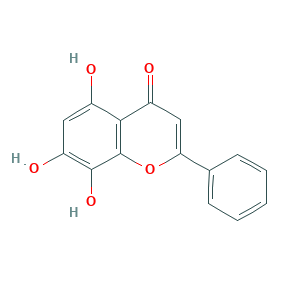 | 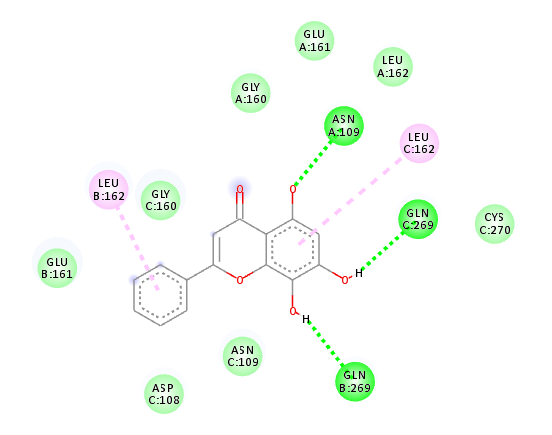 | ASN109, GLN269C, GLN269B | LEU162B, LEU162C | -7.9 |
| 47 | Pinocembrin  (68071) | 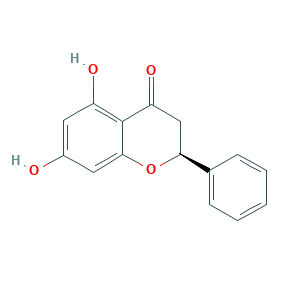 | 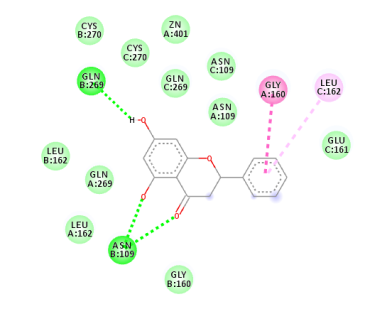 | ASN109, GLN269 | GLY160, LEU160 | -7.9 |
| 48 | Chlorflavonin  (5281606) | 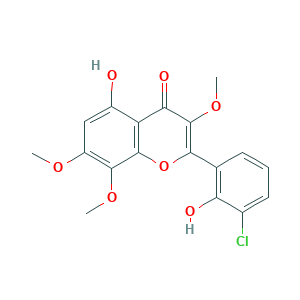 | 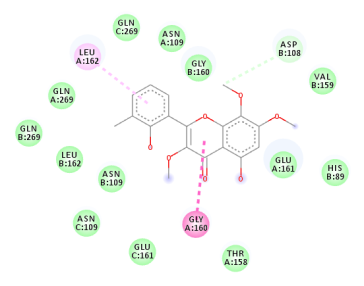 | ………… | LEU162, ASP108, GLY160 | -7.8 |
| 49 | Oroxylin A  (5320315) |  |  | …………… | LEU162, GLU161 | -7.8 |
| 50 | Sakuranetin  (73571) |  |  | ASN109B, LEU162C | GLU161A, LEU162A | -7.8 |
| 51 | 4'-Hydroxywogonin  (5322078) |  |  | GLN269, LEU162 | GLY160, LEU162 | -7.7 |
| 52 | Kolaviron  (155169) |  |  | GLN269, LEU162 | LEU162, GLY160, GLU161 | -7.7 |
| 53 | Skullcapflavone II  (124211) |  |  | ASN109C, LEU162C | LEU162C, GLY160A | -7.7 |
| 54 | Wogonin  (5281703) |  |  | ASN109C | LEU162A, LEU162B, GLU161B | -7.7 |
| 55 | Helichrysetin  (6253344) |  |  | ASN109A, ASN109C | ASN109, GLU161 | -7.6 |
| 56 | Phloretin  (4788) |  |  | ASN109A, ASN109C | LEU162, ASN109, GLU161 | -7.5 |
| 57 | Santin  (5281695) |  |  | THR158C, VAL159A | GLN269A, LEU162C, GLY160C | -7.3 |
| 58 | Isoliquiritigenin  (638278) |  |  | GLN269, GLY160 | LEU162 | -7.2 |
| 59 | Resveratrol  (445154) |  |  | GLN269B, GLN269A | GLU161, LEU162 | -7.2 |
| 60 | Hesperidin methyl chalcone  (6436550) |  |  | THR158B, VAL159, GLY160, GLY160, ASN109A, ASN109B, GLU161, THR158A | VAL159 | -7.1 |
| 61 | Pectolinarin  (168849) |  |  | ASN109 | ASN109B, ASN109A, GLY160, LEU162 | -7.0 |

*Reference compounds

**Table S2 Binding Energies and Amino Acid Interactions of Flavonoids with 3-chymotrypsin-like Protease (3CLpro) of SARS-CoV-2**

| **S/N** | **Compound**  **(PubChem CID)** | **Chemical**  **Structure** | **2D diagram of ligand interaction with amino acids** | **Hydrogen bonding–related residues** | **Non- hydrogen bonding–related residues** | **Binding Energy (Kcal/mol)** |
| --- | --- | --- | --- | --- | --- | --- |
| R1 | Lopinavir*  (92727) |  |  | GLN110 | ILE106, VAL104, PHE294, ASP295, ILE249, PRO293, HIS246, VAL202, ASP153 | -7.3 |
| R2 | Ritonavir*  (392622) |  |  | GLN110 | GLN110, ARG105, PHE103, VAL104, VAL202, PRO132, ILE200, HIS246, PHE294 | -7.1 |
| 1 | Isonymphaeol B  (10070991) |  |  | THR292, ASP295, ARG298, SER158 | THR111, PHE294, ASP153, PRO293, ILE249, VAL202, HIS246 | -8.7 |
| 2 | Baicalin  (64982) |  |  | CYS156, THR111, SER158 | PHE294, PRO293, ILE249, GLN110 | -8.3 |
| 3 | Abyssinone II  (10064832) |  |  | ASP295 | ILE249, HIS246, VAL202, ILE200, PRO293, PHE294, ARG298 | -8.2 |
| 4 | Apigetrin  (5280704) |  |  | THR111, ASP153, SER158 | PHE294, PRO293, GLN110 | -8.1 |
| 5 | Tomentin A  (71659627) |  |  | ASN151, THR111 | ILE249, PHE294 | -8.1 |
| 6 | Tomentin B  (71659628) |  |  | ASN151, THE111 | PHE294, ILE249 | -8.1 |
| 7 | Isobavachalcone  (5281255) |  |  | ASN151 | PRO293, PRO132, ILE200, HIS246 | -8.0 |
| 8 | Sanggenol A  (15233693) |  |  | SER158 | THR111, ILE249, HIS246, VAL202, PRO293, ASP153, PHE294 | -8.0 |
| 9 | Silymarin  (5213) |  |  | ----------------- | VAL202, PHE294, ASN151, ASP295, ILE249, PRO108 | -8.0 |
| 10 | Rutin  (5280805) |  |  | ASP295, GLY109 | PHE294, PRO293, THR292, ILE249 | -7.9 |
| 11 | Methoxymatteucin  (158031) |  |  | THR111 | PRO108, ILE249, PRO293, PHE294 | -7.8 |
| 12 | 3,4'-dihydroxyflavone  (145726) |  |  | THR111 | VAL202, ILE249, PRO293, THR292, PHE294 | -7.7 |
| 13 | Rhoifolin  (5282150) |  |  | SER158, GLN110, THR111, ASP295, PHE294 | SER158, PHE294, PRO108, PHE8, ASN151 | -7.7 |
| 14 | Licorice  (163463) |  |  | GLN110 | PHE294, HIS246, ILE249 | -7.6 |
| 15 | Gallocatechin gallate  (5276890) |  |  | ASN151, ASP153, LYS102 | VAL104, PHE294, ASP153 | -7.6 |
| 16 | Procyanidin  (107876) |  |  | ASP295, THR111, LYS102, ARG105 | VAL104, ASP153 | -7.6 |
| 17 | Ugonin M  (135891244) |  |  | GLY109 | VAL202, ILE249, PRO293 | -7.6 |
| 18 | Baicalein  (5281605) |  |  | ASN151, ASP295 | ARG298, ILE249, GLN110, PRO293 | -7.5 |
| 19 | Diosmetin  (5281612) |  |  | PRO108, THR111, ASN151 | VAL202, PHE294, THR292, PRO293 | -7.5 |
| 20 | Flavopiridol  (5287969) |  |  | THR111, ASP295 | PHE294, ILE249, GLN110 | -7.5 |
| 21 | Jaceosidin  (5379096) |  |  | --------------- | ILE152, PHE294, PRO108, PRO293, ILE249, GLN110 | -7.4 |
| 22 | Liquiritin apioside  (10076238) |  |  | THR198, ASP289, LYS137, GLU288 | LYS5, LYS137 | -7.4 |
| 23 | Naringenin  (932) |  |  | ASN151, THR111 | PHE294, ILE249, PRO293 | -7.4 |
| 24 | Norwogonin  (5281674) |  |  | ASP295 | THR111, PRO293, ILE249 | -7.4 |
| 25 | Oroxylin A  (5320315) |  |  | ARG298, ASN151 | PRO293, ILE249, ILE152 | -7.4 |
| 26 | Pinocembrin  (68071) |  |  | ASP295 | ASN151, PHE294, ILE249, PRO293 | -7.4 |
| 27 | Tomentin E  (71659767) |  |  | GLN110 | HIS246, PRO108, PRO293, PHE294 | -7.4 |
| 28 | Catechin  (9064) |  |  | ASP295 | GLN110, PRO293, ILE249 | -7.3 |
| 29 | Chrysin  (5281607) |  |  | ASN151, ARG298 | PHE294, ILE249, PRO293, GLN110, THR111 | -7.3 |
| 30 | Epigallocatechin gallate  (65064) |  |  | THR111, ASP295 | ASN151, ARG298, PRO293 | -7.3 |
| 31 | Fisetin  (5281614) |  |  | THR111, HIS246 | PRO293, ILE249, PHE294 | -7.3 |
| 32 | Isorhamnetin  (5281654) |  |  | THR111 | PRO108, PRO293, ILE249, PHE294 | -7.3 |
| 33 | Astragalin  (5282102) |  |  | THR111, GLN110 | GLN110, PRO293, ILE249 | -7.2 |
| 34 | Dihydroquercetin  (439533) |  |  | ASP295, THR111, HIS246 | PHE294, ILE249, PRO293 | -7.2 |
| 35 | Galangin  (5281616) |  |  | ASN151, ARG298 | ASP295, PHE294, ILE249, PRO293 | -7.2 |
| 36 | Kolaviron  (155169) |  |  | ASN151, ARG298 | ILE249, PRO293 | -7.2 |
| 37 | Lophirone A  (5488805) |  |  | LYS102, SER158, GLN110, ASP295, THR292 | VAL104 | -7.2 |
| 38 | Quercetin  (5280343) |  |  | ASP295 | ASN151, PHE294, PRO293, ILE249 | -7.2 |
| 39 | Herbacetin  (5280544) |  |  | THR111 | PHE294, GLN110, PRO293, ILE249 | -7.1 |
| 40 | Isoquercetin  (5280804) |  |  | GLN110, THR111 | GLN110 | -7.1 |
| 41 | Kaempferide  (5281666) |  |  | ASP295 | PHE294, PRO293, ILE249, GLN110 | -7.1 |
| 42 | Myricetin  (5281672) |  |  | THR111, ASN151 | ILE249, GLN110 | -7.1 |
| 43 | Phloretin  (4788) |  |  | THR111, ASN151, GLN110, ARG298 | THR292, PRO293, PHE294 | -7.1 |
| 44 | Agathisflavone  (5281599) |  |  | THR111 | PHE294, ILE249, PRO293, GLN110 | -7.0 |
| 45 | Hesperidin methyl chalcone  (6436550) |  |  | THR111, LYS102 | VAL104, PHE294 | -7.0 |
| 46 | Kaempferol-3-O-glucorhamnoside  (5318761) |  |  | GLU240, GLN110, ASN151, THR111 | HIS246, PRO293, ILE249, VAL202, PHE294 | -7.0 |
| 47 | 4'-Hydroxywogonin  (5322078) |  |  | GLN110, ARG105, SER156 | THR111, ARG298, VAL104 | -6.9 |
| 48 | Hyperin  (5281643) |  |  | THR111 | GLN110, PHE294, ILE249 | -6.9 |
| 49 | Resveratrol  (445154) |  |  | ASN151, THR111 | PHE294, PRO293, VAL202, ILE249 | -6.9 |
| 50 | Sakuranetin  (73571) |  |  | THR111 | THR111, ASN151 | -6.9 |
| 51 | Bedaquiline  (5388906) |  |  | THR175 | HIS164, GLY174, VAL148, LEU177, PRO39 | -6.8 |
| 52 | Helichrysetin  (6253344) |  |  | ASN151 | PHE294, GLN110, PRO293, PRO108 | -6.8 |
| 53 | Houttuynoid B  (57412150) |  |  | PHE294, ASN203 | ILE249, PHE294 | -6.8 |
| 54 | Isoliquiritigenin  (638278) |  |  | ASN203 | ------------- | -6.8 |
| 55 | Santin  (5281695) |  |  | ASP295, HIS246 | ILE249, PRO293, GLN110 | -6.8 |
| 56 | Wogonin  (5281703) |  |  | SER158, ILE152, GLN110 | ARG298 | -6.8 |
| 57 | Chrysosplenol C  (189065) |  |  | ASP295 | ASP295, ARG298, PHE294 | -6.7 |
| 58 | Quercitrin  (5280459) |  |  | ASP295, PRO108, GLN110 | ILE249, ASN203 | -6.6 |
| 59 | Skullcapflavone II  (124211) |  |  | THR111 | ILE152, PHE294, ILE249, PRO293, GLN110 | -6.6 |
| 60 | Chlorflavonin  (5281606) |  |  | GLN110 | PRO293, ILE249, PHE294, ASN151 | -6.5 |
| 61 | Pectolinarin  (168849) |  |  | THR111, ASN151, ASP295 | PHE8, PHE294, ARG298 | -6.1 |

*Reference compounds

Fig. S1 Graphical Summary of Pharmacokinetic Properties of Flavonoids with least Binding Energies on Binding to PLpro, and 3CLpro

The color space is the suitable physiochemical space for oral bioavailability. LIPO Lipophilicity: -0.7<XLOGP3<5.0, SIZE: 150g/mol< MW< 500g/mol., POLAR (Polarity): 20Å^2^<TPSA<130Å^2^., INSOLU (insolubility): 0<Log S (ESOL)<6., INSATU (insaturation): 0.25<Fraction Csp3<1., FLEX (Flexibity): 0< Num. rotatable bonds < 9

**Molecular Docking**

The molecular docking studies of the ligands (61 flavonoids and 2 reference compounds) and protein targets were carried out using Autodock Vina (Trott and Olson, 2010). Site specific docking were used in the studies. The grid box were from center_x = -35.261, center_y = 13.302, center_z = 25.553, size_x = 40, size_y = 40, size_z = 40 for SARS-CoV-2 papain-like protease (PLpro, PDB code 6W9C) and center_x = -16.52, center_y = -26.112, center_z = 17.524, size_x = 30, size_y = 30, size_z = 30 for 3-chymotrypsin-like protease (3CLpro, PDB code 6Y2E). The binding energies of each compound were recorded and used in rating the compounds for which PLpro and 3CLpro of SARS-CoV-2 had highest affinities. Flavonoids that displayed lower binding energies (Kcal/mol) were considered to be those for which the enzymes had higher binding affinities. Thereafter, the molecular interactions of the ligands

Trott O, Olson AJ. AutoDock Vina: Improving the Speed and Accuracy of Docking with a New Scoring Function, Efficient Optimization, and Multithreading. J Comput Chem. 2010;31(2):455–61.
